# Zap‐Pano: a Photocaged Prodrug of the KDAC Inhibitor Panobinostat

**DOI:** 10.1002/cmdc.202100403

**Published:** 2021-07-29

**Authors:** Kathrin S. Troelsen, Ewen D. D. Calder, Anna Skwarska, Deborah Sneddon, Ester M. Hammond, Stuart J. Conway

**Affiliations:** ^1^ Department of Chemistry Chemistry Research Laboratory University of Oxford Mansfield Road Oxford OX1 3TA UK; ^2^ Department of Oncology Oxford Institute for Radiation Oncology University of Oxford Oxford OX3 7DQ UK; ^3^ Department of Drug Design and Pharmacology University of Copenhagen Jagtvej 162 2100 Copenhagen Denmark

**Keywords:** Panobinostat, KDAC, prodrugs, hypoxia

## Abstract

We report the synthesis and biological evaluation of a light‐activated (caged) prodrug of the KDAC inhibitor panobinostat (Zap‐Pano). We demonstrate that addition of the 4,5‐dimethoxy‐2‐nitrobenzyl group to the hydroxamic acid oxygen results in an inactive prodrug. In two cancer cell lines we show that photolysis of this compound releases panobinostat and an unexpected carboxamide analogue of panobinostat. Photolysis of Zap‐Pano causes an increase in H3K9Ac and H3K18Ac, consistent with KDAC inhibition, in an oesophageal cancer cell line (OE21). Irradiation of OE21 cells in the presence of Zap‐Pano results in apoptotic cell death. This compound is a useful research tool, allowing spatial and temporal control over release of panobinostat.

## Graphical abstract

**Lights on, cancer out**: We report the synthesis and biological evaluation of a light‐activated (caged) prodrug of the KDAC inhibitor panobinostat (Zap‐Pano). In two cancer cell lines we show that photolysis of this compound releases panobinostat and an unexpected carboxamide analogue of panobinostat. Photolysis of Zap‐Pano causes an increase in H3K9Ac and H3K18Ac, consistent with KDAC inhibition, in an oesophageal cancer cell line (OE21). Irradiation of OE21 cells in the presence of Zap‐Pano results in apoptotic cell death.

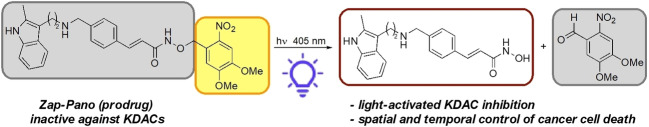

## Introduction

Protein post‐translational modifications (PTMs) add a layer of complexity to the proteome and provide mechanisms to fine‐tune protein function.^[1]^ Lysine acetylation is a well‐established PTM^[2]^ that is found in over 3600 locations on over 1750 proteins throughout the cellular environment.[^3^, ^4^] Recently, there has been a particular focus on lysine acetylation in histone proteins, which play a key role in the organisation of DNA in chromatin. Acetylation leads to the formation of an amide, which removes the positive charge from the ϵ‐nitrogen atom of lysine, resulting in a weaker association with negatively charged DNA, and the formation of euchromatin. This more relaxed form of chromatin is associated with transcriptional activation. Lysine acetylation state is regulated by histone/lysine acetyl transferase (H/KATs) enzymes, which transfer an acetyl group from AcCoA to lysine, and the mechanistically distinct sirtuin and Zn^2+^‐dependent histone/lysine deacetylases (H/KDACs) that remove this mark.[^5^, ^6^]

Bromodomains are protein modules that bind to acetyl‐lysine (KAc) residues and in so doing mediate protein‐protein and protein‐chromatin interactions enabling the assembly of transcription complexes.[^7^, ^8^, ^9^, ^10^]

Many examples exist of KDAC dysregulation being linked to oncogenic states in tissues throughout the body.^[11]^ These observations have led to significant interest in molecules that can inhibit these enzymes, culminating in the clinical approval of five pan‐KDAC inhibitors to date (belinostat, chidamide, panobinostat (Pano, **2**), romidepsin, and vorinostat).^[11]^

All KDAC inhibitors possess a group that binds to the Zn^2+^ ion found in the enzyme active site, which mimics KAc binding in the active site, and inhibits enzyme function. For belinostat, Pano and vorinostat this moiety is an hydroxamic acid. In addition to Zn^2+^, this group can bind to other metals or inhibit other off‐target enzymes. Hydroxamic acids are also polar and can hinder the diffusion of KDAC inhibitors into cells and organs, for example, the brain. KDACs are also broad epigenetic remodellers that affect the transcription of many genes, in both healthy tissues and tumours.

For these reasons, KDAC inhibitors have a number of documented side effects including anaemia, neutropenia, thrombocytopenia, fatigue, diarrhoea, nausea and vomiting.[^13^, ^14^] In recent years, attempts have been made to increase the selectivity of these inhibitors and therefore mitigate these unwanted effects. Work to achieve this has included both designing selective inhibitors of a given KDAC enzyme, or developing prodrugs that selectively release inhibitors in a target tissue.[^15^, ^16^, ^17^, ^18^] Prodrugs of KDAC inhibitors have been developed that are activated by hydrolysis,^[19]^ thiols,^[13]^ reactive oxygen species,^[20]^ hydrogen peroxide or peroxynitrite,^[21]^ esterases,[^22^, ^23^] transition metals,^[24]^ and light.[^25^, ^26^, ^27^, ^28^, ^29^] We have recently reported prodrugs of the KDAC inhibitors vorinostat (SAHA)^[30]^ and Pano^[12]^ that are selectively released in hypoxia.

**Figure 1:**
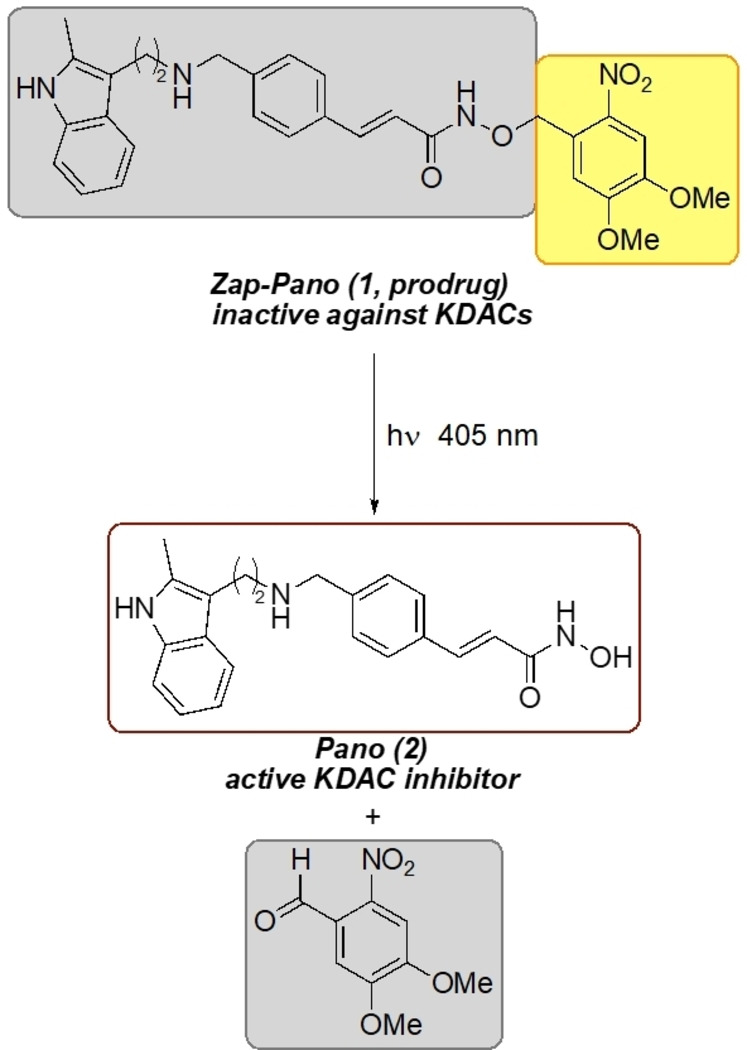
The light‐activated derivative of Pano (**2**), Zap‐Pano (**1**), is photolysed to release the KDAC inhibitor Pano (**2**).

We have previously used a caging approach to develop photolabile precursors of capsaicin,[^31^, ^32^] the mitochondrial uncoupler AG10,^[33]^ and the neurotransmitters glutamate and GABA.^[34]^ This technique provides temporal and spatial control over the release of the active component. Building on our work developing a hypoxia‐activate prodrug of Pano,^[12]^ here we report the design, synthesis, and initial validation of a light‐activated prodrug of Pano, Zap‐Pano (**1**, Figure 1). This compound releases Pano with temporal control in the oesophageal cancer cell line, OE21; it will be a useful research tool for those studying lysine acetylation and has the potential to allow the release of Pano while minimising off‐target effects.^[35]^

## Results and Discussion

### Chemistry

**Scheme 1:**
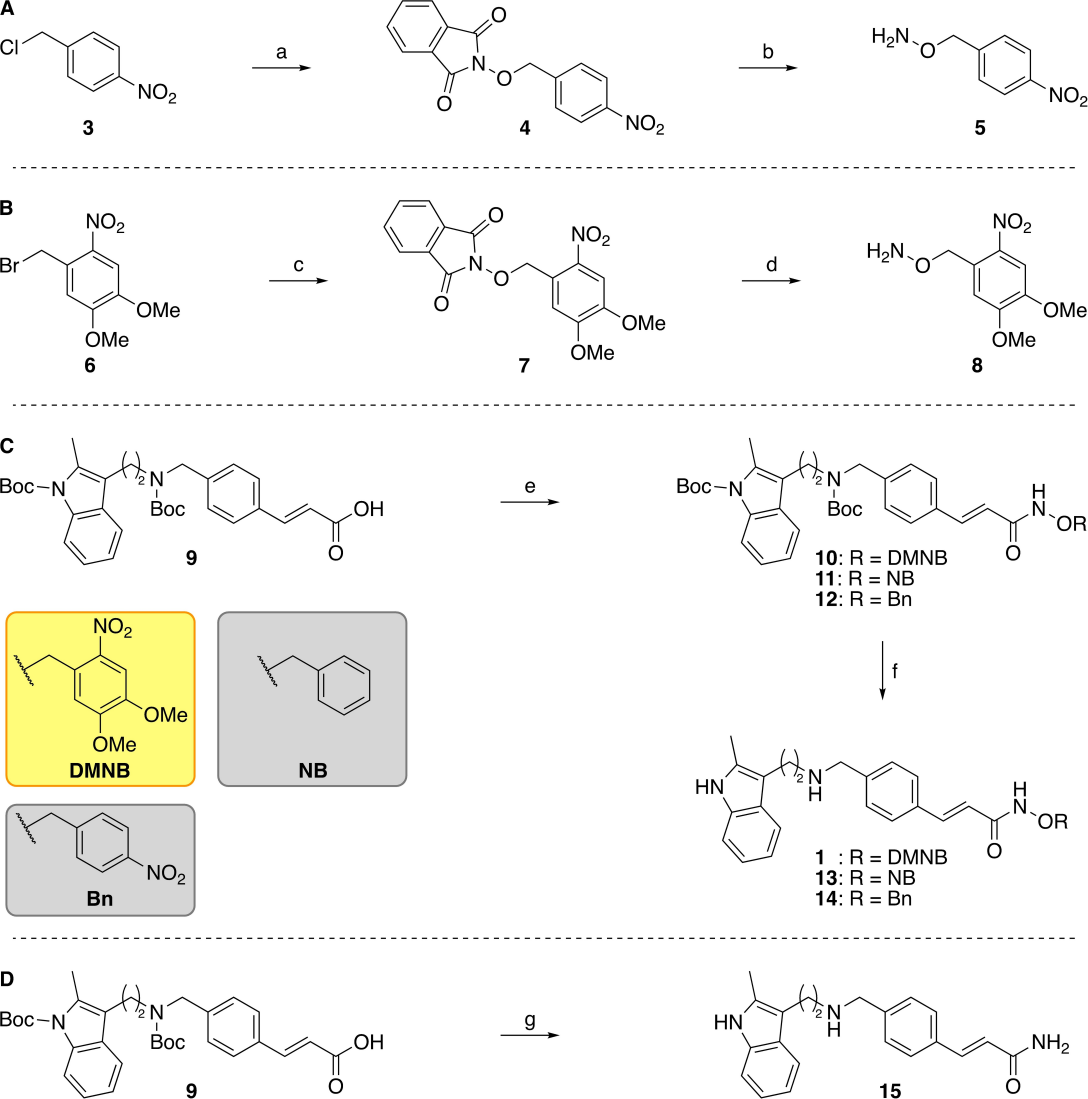
Synthesis of Zap‐Pano (**1**) and the benzylated Pano derivatives **13** and **14**, and the amide **15**. Reagents and conditions: (**A**) (a) *N*‐Hydroxyphthalimide, DIPEA, DMF, 0 °C to rt, 2 h, 76–84 %, n=2, (b) N_2_H_4_⋅H_2_O, CH_2_Cl_2_, rt, 2 h, 72–95 %, n=4.^[12]^ (**B**) (c) *N*‐Hydroxyphthalimide, DIPEA, DMF, 70 °C, 2 h, 88 %, n=1; (d) N_2_H_4_⋅H_2_O, CH_2_Cl_2_, rt, 3 h, 75–99 %, n=2. (**C**) (e) RONH_2_, PyBOP, NEt_3_, THF, rt, 18 h, **10**, R=DMNB, 62–74 %, n=2, RONH_2_, PyBOP, NEt_3_, THF, rt, 18 h, **11**, R=NB, 71–92 %, n=2, RONH_2_, PyBOP, NEt_3_, THF, rt, 18 h, **12**, R=Bn, 73 %, n=1; (f) TFA, TIPS−H, CH_2_Cl_2_, rt, 1 h, **1**, R=DMNB, 51–65 %, n=2, TFA, TIPS−H, CH_2_Cl_2_, rt, 1 h, **13**, R=NB, 68–69 %, n=2, TFA, TIPS−H, CH_2_Cl_2_, rt, 1 h, **14**, R=Bn, 79 %, n=1. (**D**) (g) (i) PyBOP, NEt_3_, NH_4_Cl, THF, rt, 18 h, 84 %, n=1; (ii) TFA, TIPS−H, CH_2_Cl_2_, rt, 1 h, 65 %, n=1.

We, and others, have previously demonstrated that substitution on the hydroxamic acid oxygen atom is an effective strategy for developing prodrugs of KDAC inhibitors. This modification can prevent the interaction between the compound and the active site Zn^2+^ ion, leading to an inactive compound.[^12^, ^30^] Therefore, we adopted this approach to develop light‐activated derivatives of Pano and selected the 4,5‐dimethoxy‐2‐nitrobenzyl group as the photo‐labile component (Scheme 1).– [^33^, ^36^, ^37^] In addition, we also employed two negative control compounds a 4‐nitrobenzyl analogue (NB, **13**) and an unsubstituted benzyl analogue (Bn, **14**), which should not photorelease Pano.

The synthesis of Pano was carried out as has been previously published.[^12^, ^38^] The photolabile DMNB derivative of Pano (Zap‐Pano, **1**) and both control compounds were synthesised from the substituted hydroxylamines using a route previously developed within the group (Scheme 1).[^12^, ^30^] Briefly, the nitrobenzyl halides (**3** and **6**) were reacted with *N*‐hydroxyphthalimide, before deprotection with hydrazine, to give the substituted hydroxylamines (**5** and **8**). Both of the synthesised hydroxylamines (**5** and **8**), and the commercially available *O*‐benzylhydroxylamine, were then coupled to the di‐Boc protected core fragment of Pano (**9**) using PyBOP. The Boc groups were removed using trifluoroacetic acid, to yield Zap‐Pano (**1**), and the two control compounds (**13** and **14**). We also synthesised the carboxamide analogue of Pano, **15**, as this compound was detected as a by‐product in the photolysis reaction (*vide infra*).

### Zap‐Pano does not inhibit KDAC enzymes

Analysis of DMNB‐Pano in a commercial KDAC inhibition assay showed that this compound does not inhibit KDAC1‐9 or KDAC11 to any significant degree (Table 1). This is consistent with our previous work on prodrugs of SAHA and Pano,[^12^, ^30^] and encouraged us to progress Zap‐Pano for further analysis.

**Table 1:**
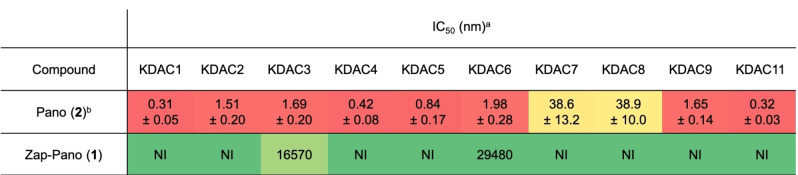
Zap‐Pano shows little KDAC inhibition *in vitro*.

IC_50_ values (nM) for Pano and Zap‐Pano against KDAC1‐9 and KDAC11. The colour scale represents a heat map, with ‘hot’ colours showing effective enzyme inhibition. [a] Data obtained by Reaction Biology Corporation, NI=no inhibition observed at concentrations up to 10 μM. [b] Data taken from Arts *et al*.^[39]^

### Photolysis of Zap‐Pano releases Pano in PBS buffer

**Figure 2:**
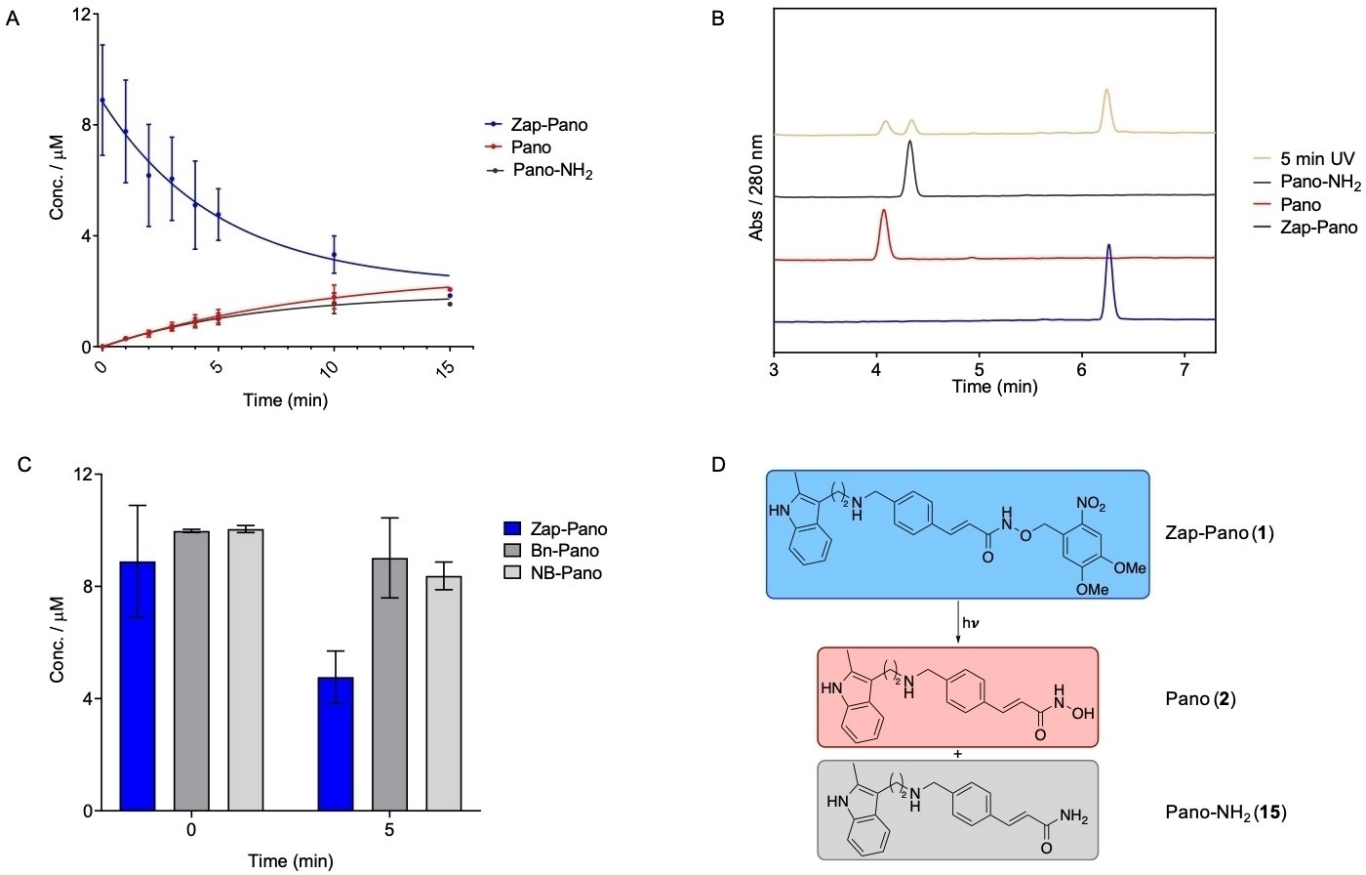
Photolysis of Zap‐Pano in PBS buffer. (**A**) Zap‐Pano 10 μM in PBS was treated with UV‐light (405 nm) for up to 15 min, aliquots were taken at desired time points and analysed by HPLC (10 μL injections) to determine the concentration of Zap‐Pano, Pano and Pano‐NH_2_. (**B**) Overlaid HPLC traces of Zap‐Pano (blue), Pano (red), Pano‐NH_2_ (black) Zap‐Pano treated with UV light for 5 min (green). Retention times are 6.25 min for Zap‐Pano, 4.08 min for Pano and 4.33 min for Pano‐NH_2_. (**C**) Zap‐Pano, Bn‐Pano (**14**) and NB‐Pano (**13**) were irradiated with UV‐light for 5 minutes then the products quantified using HPLC analysis. (**D**) Chemical structures of Zap‐Pano (**1**), Pano (**2**) and the unexpected by‐product of photolysis Pano‐NH_2_ (**15**).

The uncaging of Zap‐Pano and release of Pano was first evaluated in PBS buffer. Upon irradiation with light of wavelength 405 nm for 15 min, we observed approximately 20 % Pano being released. However, we were surprised to also observe approximately 20 % of an amide derivative of Pano by‐product (Pano‐NH_2_, **15**, Figure 2D), which was formed during irradiation (Figure 2A, B). The structure of **15** was confirmed using LCMS analysis (see Supporting Information) and synthesis of an authentic sample for comparison (Scheme 1D). To determine whether the by‐product was formed as a result of photolysis, or resulted from instability of Zap‐Pano in PBS, the two control compounds, NB‐Pano (**13**) and Bn‐Pano (**14**), were treated with UV‐light and analysed using HPLC.

**Scheme 2:**
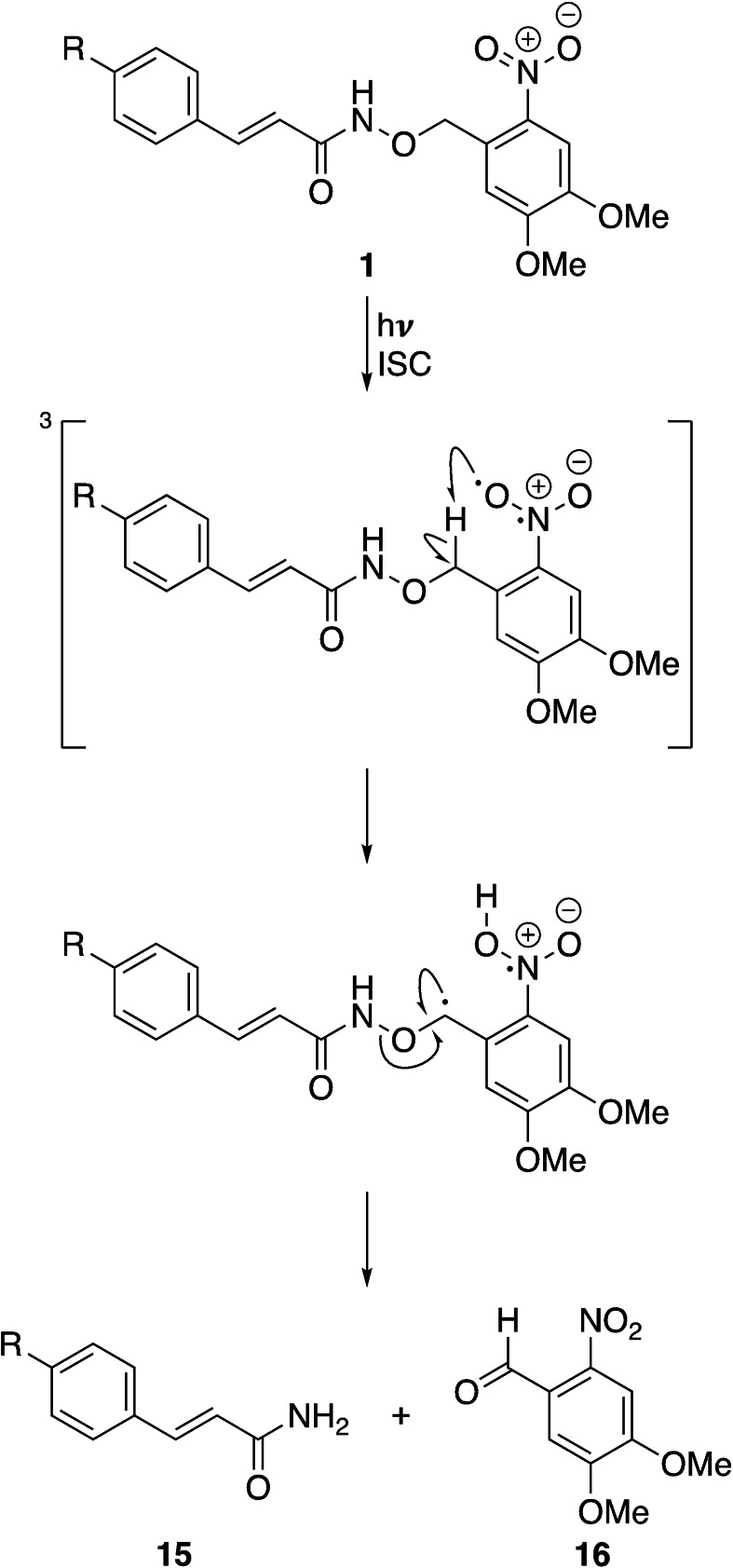
Proposed mechanism for the formation of the carboxamide derivative of Pano, Pano‐NH_2_ (**15**). ISC=intersystem crossing.

Interestingly, both compounds were stable to UV irradiation, and did not release any by‐products that could be detected with HPLC (Figure 1C). Based on these observations, a plausible mechanism for the formation of **15** is shown in Scheme 2. Given that this product is only formed when **1** is irradiated with light, we suggest that the first step is excitation to the singlet state, followed by intersystem crossing to give the triplet state, as is the case for the expected photolysis pathway.^[37]^ However, the relatively weak N−O bond (201 kJ mol^−1^) of the hydroxamic acid presents an alternative pathway for the radical reaction to proceed. We suggest that the benzylic radical can react to form the nitrobenzaldehyde **16**, and the amide product **15**, as shown in Scheme 2.

### Zap‐Pano releases Pano in response to UV‐treatment of cancer cells

**Figure 3:**
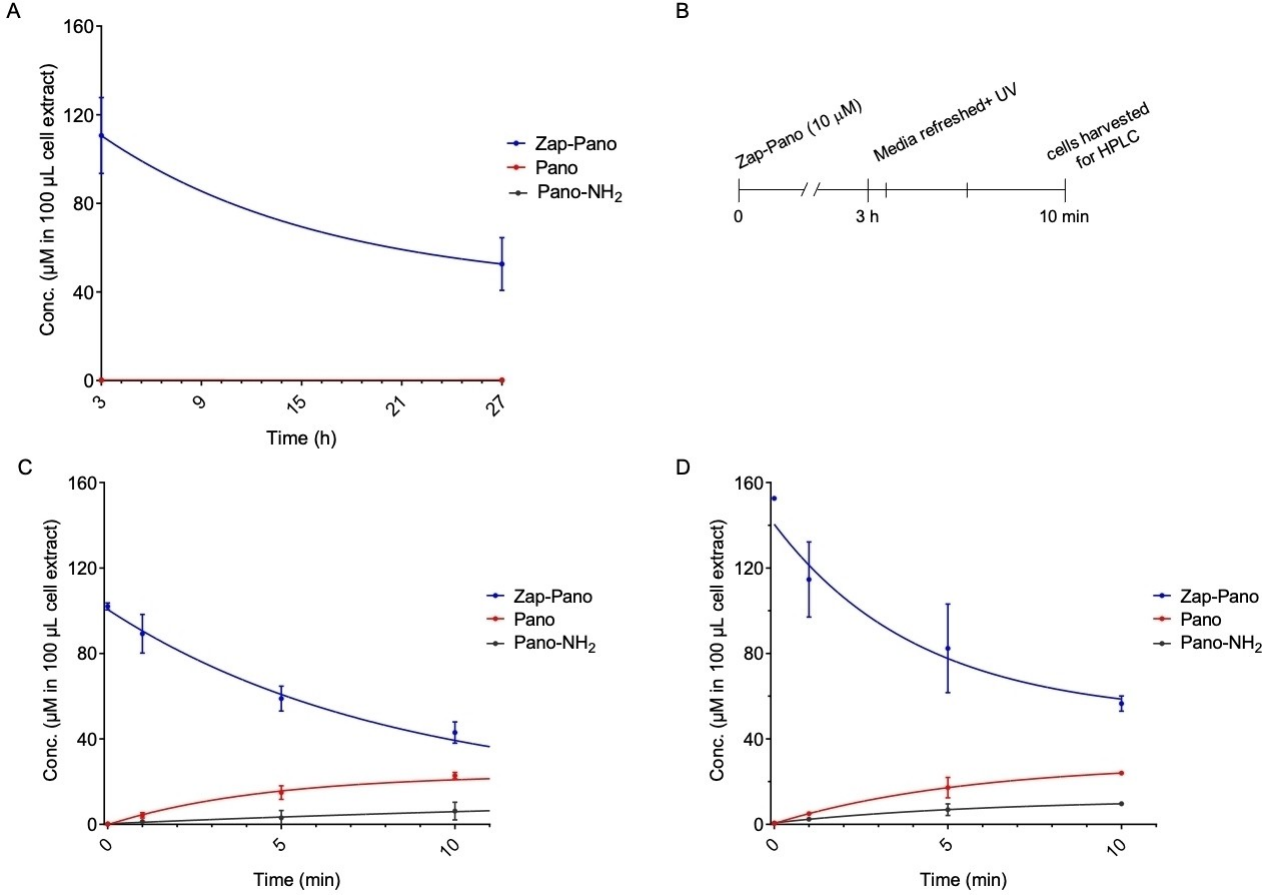
Photo‐uncaging of Zap‐Pano in cancer cell lines. (**A**) OE21 cells were treated with Zap‐Pano (10 μM) and incubated for 3 or 27 h Cell lysis was performed in MeCN:MeOH (1 : 1) with a fixed volume of 100 μL. Cell lysate was analysed using HPLC to determine the concentration of Zap‐Pano, Pano and Pano‐NH_2_. Quantification was made using a calibration curve for Zap‐Pano, Pano and Pano‐NH_2_ (n=3). (**B**) A schematic representation of the photo‐uncaging experiments is shown. (**C**) OE21 cells and (**D**) HCT116 cells were treated with Zap‐Pano (10 μM). After 3 h, the media containing Zap‐Pano was removed and replaced with fresh media. The cells were then exposed to UV‐light for the time periods indicated (0–10 minutes). Cell lysis was performed in MeCN:MeOH (1 : 1) with a fixed volume of 100 μL. Cell lysates were analysed using HPLC to determine the concentration of Zap‐Pano, Pano and Pano‐NH_2_.

Next, we investigated the uptake and light‐activation of Zap‐Pano. We chose to use two cancer cell lines OE21 (oesophageal) and HCT116 (colorectal) as both originate from cancer types that are accessible with a UV light i. e., the oesophagus and colon/rectum. To demonstrate that Zap‐Pano is cell permeable, the cells were treated with 10 μM Zap‐Pano followed by HPLC analysis to determine the cellular concentration of Zap‐Pano, Pano and Pano‐NH_2_. Pleasingly, Zap‐Pano was found to be cell permeable and stable over a 24‐hour period (Figure 3A). Most importantly, when OE21 or HCT116 cells were treated with Zap‐Pano (10 μM for 3 hours) followed by UV treatment, the levels of Zap‐Pano decreased and there was a corresponding increase in Pano (Figure 3B, C, D). The released Pano was still detectable 24 hours after UV treatment; 1.65±0.8 nM in OE21 cells (data not shown).

### Zap‐Pano has a UV‐dependent effect on H3K9Ac and H3K18Ac levels

**Figure 4:**
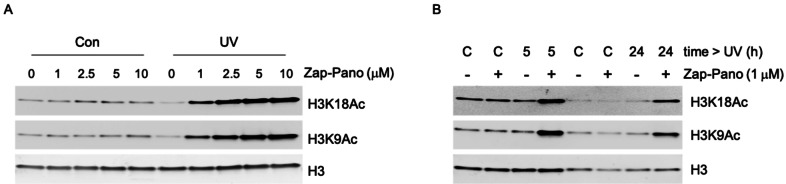
OE21 cells treated with Zap‐Pano and exposed to UV light have increased H3 K18 and H3 K9 acetylation. (**A**) OE21 cells were treated with the doses of Zap‐Pano indicated (0–10 μM) for 3 h and were then either kept in the dark (Con) for 24 h or treated with UV light for 5 min followed by incubation for 24 h. The levels of H3 K9 and H3 K18 acetylation are shown, H3 was included as a loading control. (**B**) OE21 cells were treated with 1 μM Zap‐Pano for 3 h followed by UV light (5 min) or no light (labelled C), the cells were allowed to grow for 5 h or 24 h before lysing and immunoblotting for H3K9Ac and H3K18Ac.

Numerous studies have confirmed that nanomolar doses of Pano lead to increases in acetylation of histone H3 including at lysines 9 and 18.[^40^, ^41^, ^42^, ^43^] To determine the effects of Zap‐Pano on histone acetylation, OE21 cells were treated with Zap‐Pano for 3 hours then exposed to UV (5 minutes) before being returned to normal culture conditions (24 hours). Cells were then harvested, and western blotting carried out for H3K18Ac and H3K9Ac (Figure 4A). Importantly, in the absence of exposure to UV light there was no change in H3K18Ac or H3K9Ac, confirming that the prodrug does not act as a KDAC inhibitor in cells. However, after UV exposure a clear dose dependency was observed for both acetylation marks. Having determined that a dose of 1 μM Zap‐Pano was sufficient to increase H3K18Ac and H3K9Ac, we further investigated the time after UV exposure required to observe these changes. OE21 cells were again treated with Zap‐Pano for 3 hours followed by UV exposure. The cells were then harvested after 24 hours (as previously) or after 5 hours. A clear increase in H3K18Ac and H3K9Ac was observed at both 5 and 24 hours after UV exposure (Figure 4B).

### Zap‐Pano leads to loss of cell viability in a UV‐dependent manner

**Figure 5:**
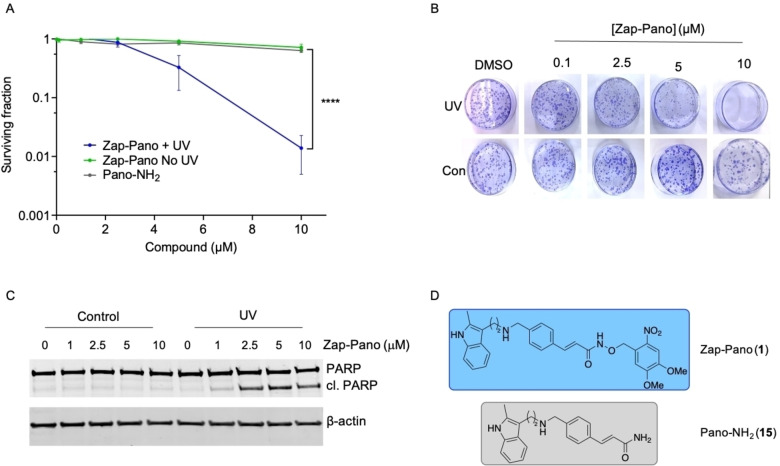
Zap‐Pano leads to loss of cell viability in a UV‐dependent manner. (**A**) Colony survival assay of OE21 cells treated with Zap‐Pano and Pano‐NH_2_.^[44]^ Cells were treated at the concentrations of Zap‐Pano shown (grey), Zap‐Pano followed by 5 min exposure to UV light (purple), and Pano‐NH_2_ (grey). Mean ± SEM of three independent experiments performed in triplicate are shown. Statistical significance was determined using two‐way ANOVA, ****p<0.0001. (**B**) Representative images from part A are shown. (**C**) OE21 cells were exposed to the doses indicated of Zap‐Pano for 3 h followed by UV or mock treatment (Control). Cells were harvested 24 h after UV and western blotting carried out. β‐actin is shown as a loading control. (**D**) The chemical structures of Zap‐Pano (**1**) and Pano‐NH_2_ (**15**).

Next, we determined the toxicity of Zap‐Pano (**1**, Figure 5D) both with and without UV‐exposure and compared to Pano‐NH_2_ (**15**, Figure 5D). OE21 cells were exposed to a range of doses of Zap‐Pano (0–10 μM) or Pano‐NH_2_ and a colony survival assay carried out (Figure 5A, B).

As expected, Pano‐NH_2_ and Zap‐Pano in the absence of UV light did not significantly affect clonogenic potential of OE21 cancer cells. However, when Zap‐Pano was combined with UV light treatment, a significant decrease in OE21 colony forming and survival was observed. As Pano has been shown to promote apoptosis,^[41]^ we investigated whether Zap‐Pano could also cause apoptotic cell death in the presence of UV light. To investigate this hypothesis western blotting was carried out for PARP cleavage, a well‐characterised marker of apoptosis (Figure 5C). PARP cleavage was observed only in the cells treated with Zap‐Pano that were exposed to UV. Together these results indicate that Zap‐Pano has the potential to decrease cancer cell viability in a light‐dependent manner.

## Conclusion

In conclusion, we have designed and synthesised a DMNB‐derivative of Pano, Zap‐Pano **1**. We have shown that this compound does not inhibit KDAC enzymes *in vitro* or in cells. Photolysis of Zap‐Pano causes release of Pano and an unexpected amide derivative **15**. Photolysis of Zap‐Pano in the OE21 cell line causes an increase in acetylation at H3 K9 and K3 K18, consistent with light‐dependent KDAC inhibition by the released Pano. Photolysis of Zap‐Pano in OE21 cells also causes cell death, and an increase in PARP cleavage, which indicates that this results from KDAC inhibition and subsequent apoptosis. These data show that Zap‐Pano compound is a powerful research tool that places KDAC inhibition, and therefore lysine acetylation state, under the control of light, providing temporal and spatial control over their inhibition. As the cellular testing of this compound was conducted using cell lines derived from cancers that are accessible to light, our data suggest that Zap‐Pano has the potential to be used in photodynamic therapy, where the release of Pano is controlled by light, minimising the effects of Pano on non‐cancerous tissue.

## Experimental Section

### Biology Experimental section

**Cell lines**. OE21 (oesophageal squamous carcinoma, PHE) and HCT116 (colorectal carcinoma, gift from Professor Bert Vogelstein, Johns Hopkins Medical School, USA) cancer cells were cultured in RPMI and DMEM medium, respectively, supplemented with 10 % FBS, penicillin (100 μg/mL) and streptomycin (100 μg/mL). Cells were grown at 37 °C in a humidified atmosphere of 5 % of CO_2_. Cells were routinely tested for mycoplasma and found to be negative.

**LED photolysis experiments** were performed with 405 nm LED, 800 mA, 3.3 V, WL‐SUMW SMT Ultraviolet Ceramic Waterclear, Würth Elektronik.

**High‐performance liquid chromatography** (HPLC) analysis was carried out using HPLC (Waters 2695, Watford, UK) with a photodiode array detector (Waters 2996). Separation was achieved using a HiChrom RPB column (C18, 5 μm, 3.2×100 mm) maintained at 35 °C and flow rate of 0.5 mL/min. The system was employed with 0.01 % *v/v* TFA in water as eluent A and acetonitrile as eluent B. Samples were injected in methanol, water or acetonitrile. The method was used as shown in Table 2.

**Table 2:** HPLC conditions.

[A]	[B]	Time [Min]	Slope
80 %	20 %	0	0
30 %	70 %	6	1
80 %	20 %	6.1	0
80 %	20 %	10	0

**Liquid chromatography mass spectrometry** (LCMS) was carried out using an Agilent 1260 Infinity II system equipped with a vial sampler, diode array detector, fraction collector and single quadrapole LC/MSD. A sample (10 μL) of Pano‐NH_2_ (**15**) spiked with Pano acid was injected onto an Agilent Zorbax RRHT StableBond SB−C18 column (2.1×50 mm, 1.8 μM) at 40 °C following the specified gradient profile below. The identity of Pano‐NH_2_ (**15**, 1.071 min) and Pano acid (2.263 min) was confirmed using mass spectrometry. The method was used as shown in Table 3.

**Table 3:** LCMS conditions.

Time (min)	Flow rate [mL/min]	%A H_2_O+0.1 % *v/v* formic acid	%B MeCN+0.1 % *v/v* formic acid
0	0.5	80	20
6	0.5	30	70
6.1	0.5	80	20
10	0.5	80	20

**Photorelease experiments** Zap‐Pano (10 μM in PBS, 4 mL) was placed in a 6 cm plastic dish under the LED (405 nm, 800 mA). Aliquots (50 μL) were taken at the indicated time points and diluted in MeCN (50 μL). Quantification was made using a calibration curve for Zap‐Pano, Pano, and Pano‐NH_2_ measuring absorbance at 280 nm, and are not scaled from the HPLC injection volume. OE21 or HCT116 cells (0.5×10^6^, 6 cm dish, 4 mL media) were seeded overnight and treated with Zap‐Pano (10 μM) for 3 h allowing sufficient time for the drug to accumulate within cells. Next, the drug containing medium was removed and replaced with drug‐free medium. Cells were then placed under the LED for the indicated time. Immediately after UV treatment cells were washed twice with PBS, scraped into 1 mL of PBS, and centrifuged at 3000 *g* for 5 minutes. Cell pellets were lysed in ice‐cold MeOH:MeCN (1 : 1, 100 μL), briefly sonicated and cell debris were removed by centrifugation at 13300 *g* (15 min). Resulting supernatants were analysed by HPLC (10 μL injections) to determine the concentration of Zap‐Pano, Pano and Pano‐NH_2_. Quantification was performed using a calibration curve for Zap‐Pano, Pano, and Pano‐NH_2_ at 280 nm and were not scaled from the HPLC injection volume.

**Immunoblotting**. Following Zap‐Pano/UV treatment cells were washed in PBS, lysed in UTB buffer (9 M urea, 75 mM Tris‐HCl pH 7.5, 0.15 M β‐mercaptoethanol). Cell lysates were briefly sonicated, centrifuged for 15 mins at 13500 rpm, and the supernatant was collected for western blotting. 50 μg of total proteins were separated by SDS‐gel electrophoresis and electroblotted onto nitrocellulose membranes, which were subsequently probed with the following primary antibodies: anti‐PARP (9542 Cell Signaling), anti‐H3K18Ac (9675 Cell Signaling), anti‐H3K9Ac (949S Cell Signaling), anti‐H3 total (3638S Cell Signaling), and anti‐β‐actin (sc‐69876 Santa‐Cruz biotechnology). Secondary antibodies were IRDye® 680RD Goat anti‐Mouse IgG (H+L) and IRDye® 800CW Donkey anti‐Rabbit IgG (H+L) from LI‐COR Biosciences. Odyssey IR imaging technology (LI‐COR Biosciences) was used for imaging.

**Colony survival assay**. OE21 cells were seeded at low density in 3.5 cm Petri dishes (500‐1000 cells/dish), allowed to attach and treated with Zap‐Pano for 3 h. The drug containing media was removed and replaced with drug‐free medium, cells were irradiated for 5 minutes and then the media was again replaced with fresh media. Cells were incubated in normal conditions for 6–10 days to form colonies (>50 cells). Colonies were fixed in 75 % methanol and stained with 0.5 % crystal violet. Colonies were counted manually to quantify the surviving fraction.

### Chemistry Experimental Section

#### General chemistry procedures

**Chemicals** were purchased from Acros Organics, Alfa Aesar, Apollo Scientific, Fisher Scientific, Fluka, Fluorochem, Merck or Sigma Aldrich and were used without further purification. Where appropriate and if not otherwise stated, all non‐aqueous reactions were carried out under an inert atmosphere of argon, using flame‐dried glassware. **Anhydrous solvents** were obtained under the following conditions: THF, acetonitrile, dichloromethane, diethyl ether and DMF were dried by passing them through a column of active basic alumina according to Grubbs’ procedure and stored over activated 3 Å molecular sieves under argon.^[45]^ Anhydrous methanol and ethanol was purchased from Sigma Aldrich UK in SureSeal™ bottles and used without further purification. **Analytical thin layer chromatography (TLC)** was performed on normal phase Merck silica gel 60 F254 aluminium‐supported thin layer chromatography sheets. Spots were visualised by either absorption under UV light (254 nm), exposure to iodine vapor or thermal development after dipping into a solution of ammonium molybdate in sulfuric acid, an aqueous solution of potassium permanganate or an ethanolic solution of ninhydrin. Reaction progress was monitored at appropriate times using TLC analysis. Normal phase silica gel flash column chromatography was performed manually using Geduran Silica gel 60 (40–63 μm) under a positive pressure of compressed nitrogen or on a Biotage SP1 automated column chromatography system using KP‐Sil® SNAP Flash Silica Cartridges. ^**1**^ **H NMR spectra** were recorded on a Bruker AVIIIHD 400 (400 MHz) a Bruker AVII 500 with dual ^13^C(^1^H) cryoprobe (500 MHz) or a Bruker AVIIIHD 500 (500 MHz) spectrometer with the stated solvents as a reference for the internal deuterium lock. Chemical shifts are reported as δ_H_ in parts per million (ppm) relative to tetramethyl silane (TMS). The spectra are calibrated using the solvent peak with the data provided by Fulmer *et al*.^[46]^ Identical proton coupling constants are averaged in each spectrum and reported to the nearest 0.1 Hz. The coupling constants were determined by analysis using Bruker TopSpin software (versions 3.2 and 4.0) or Mestrenova software (version 11). ^1^H spectra were assigned using 2D NMR experiments including ^1^H‐^1^H COSY, ^13^C‐^1^H HSQC and ^13^C‐^1^H HMBC. ^**13**^ **C NMR spectra** were recorded on a Bruker AVIIIHD 400 (101 MHz) or a Bruker AVII 500 with dual ^13^C(^1^H) cryoprobe (126 MHz) spectrometer in the stated solvents with broadband proton decoupling and an internal deuterium lock. Chemical shifts are reported as δ_C_ in parts per million (ppm) relative to tetramethyl silane (TMS). The spectra are calibrated using the solvent peak with the data provided by Fulmer *et al*.^[46]^ The shift values of resonances are quoted to 1 decimal place unless peaks have similar chemical shifts, in which case 2 decimal places are used. ^13^C spectra were assigned using 2D NMR experiments including HSQC and ^13^C‐^1^H HMBC. **Electrospray ionisation (ESI) mass spectra** were acquired using an Agilent 6120 Quadrupole spectrometer or Waters LCT Premier spectrometer, operating in positive or negative mode, as indicated, from solutions of MeOH or MeCN. Chemical ionisation (CI) mass spectra were acquired using a Waters GCT spectrometer. MS data was processed using Mestrenova software (version 11). *m/z* values are reported in Daltons and followed by their percentage abundance in parentheses. **Accurate mass spectra** were obtained using Bruker μTOF spectrometer. *m/z* values are reported in Daltons. When a compound was not observed by LRMS, only HRMS is quoted. **Melting points** were determined using either a Griffin capillary tube melting point apparatus or a Kofler hot stage and are uncorrected. The solvent(s) from which the sample was crystallised is given in parentheses. **Infrared (IR) spectra** were obtained either from neat samples, either as liquids or solids, or as a thin film using a diamond ATR module. The spectra were recorded on a Bruker Tensor 27 spectrometer. Absorption maxima are reported in wavenumbers (cm^−1^). Only the main, relevant peaks have been assigned. **UV/vis spectra** were obtained as 10 μM solutions in water on a Shimadzu UV‐1800 spectrophotometer and raw data were processed in GraphPad Prism 7.

**Purity** of all novel compounds was determined using analytical high‐performance liquid chromatography (HPLC; Table 4) on a PerkinElmer Flexar system with a Binary LC Pump and UV/vis LC Detector. All biologically tested compounds were of >95 % purity as determined by HPLC. For determination of compound purity on reversed phase (RP) 2–10 μL of sample was injected a Dionex Acclaim® 120 column (C18, 5 μm, 12 Å, 4.6×150 mm). The gradient profile, unless otherwise stated is shown in Table 3. **Method A** refers to the solvents shown without modifiers. **Method B** refers to the solvents shown with the addition of 0.1 % *v/v* TFA.

**Table 4:** HPLC conditions.

[A]	[B]	Time [Min]	Slope
95 %	5 %	5	0
5 %	95 %	10	1
5 %	95 %	5	0

### Experimental methods

#### *N*‐Phthalimido‐*O*‐(4, 5‐dimethoxy‐2‐nitrobenzyl)‐hydroxylamine (7)

The reaction, work‐up and purification were carried out in the dark as far as possible. *N*,*N*‐Diisopropylethylamine (345 μL, 1.98 mmol, 1.2 eq) was added to a stirred solution of *N*‐hydroxyphthalimide (269 mg, 1.65 mmol, 1.0 eq) in *N*,*N*‐dimethylformamide (8 mL). 4,5‐Dimethoxy‐2‐nitrobenzyl bromide (**6**, 500 mg, 1.81 mmol, 1.1 eq) was added and the solution was heated to 70 °C for 2 h. The reaction solution was cooled to rt, diluted with ethyl acetate (200 mL), and washed with aqueous 0.5 M lithium chloride (4×200 mL) then dried (MgSO_4_), filtered, and concentrated *in vacuo*. Crystallisation from hot ethanol yielded the title compound (**7**) (520 mg, 88 %) as a colourless solid. *R_f_* 0.40 (50 % ethyl acetate:petroleum ether); mp 188–190 °C (from EtOH); *ν*_max_ (thin film)/cm^−1^ 2950, 1740, 1525, 1498, 1281, 1066; ^1^H NMR (400 MHz, CDCl_3_) δ_H_ 7.88–7.81 (2H, m), 7.80–7.73 (2H, m), 7.70 (1H, s), 7.61 (1H, s), 5.68 (2H, s), 4.08 (3H, s), 3.96 (3H, s); ^13^C NMR (101 MHz, CDCl_3_) δ_C_ 163.5, 153.9, 148.5, 139.8, 134.8, 128.9, 126.4, 123.8, 110.5, 108.0, 76.3, 56.8, 56.5; HRMS *m/z* (ESI^+^) Found: 381.06925, C_17_H_14_N_2_O_7_Na requires [M+Na]^+^ 381.06932; LRMS *m/z* (ESI^+^) 381 (100 %, [M+Na]^+^), 196 (30 %); HPLC Method A, Retention time ‐ 9.5 min, 92 %.

#### *O*‐(4, 5‐Dimethoxy‐2‐nitrobenzyl)‐hydroxylamine (8)

The reaction, work‐up and purification were carried out in the dark as much as possible. Hydrazine monohydrate (65 % *v/v* in water) (167 μL, 2.23 mmol, 4.0 eq) was added to a suspension of *N*‐phthalimido‐*O*‐(4’,5’‐dimethoxy‐2’‐nitrobenzyl)‐hydroxylamine (**7**) (200 mg, 0.559 mmol, 1.0 eq) in dichloromethane (11 mL) and stirred at rt for 3 h. The suspension was diluted with dichloromethane (20 mL) and filtered, then concentrated *in vacuo* to yield the title compound (**8**) (126 mg, 99 %) as a yellow solid which was used without further purification. *R_f_* 0.10 (50 % ethyl acetate:petroleum ether); mp 82–84 °C (from dichloromethane/hexane); ^1^H NMR (400 MHz, CDCl_3_) δ_H_ 7.68 (1H, s), 7.12 (1H, s), 5.58 (2H, br s), 5.10 (2H, s), 4.00 (3H, s), 3.95 (3H, s); ^13^C NMR (101 MHz, CDCl_3_) δ_C_ 153.7, 147.9, 140.2, 129.8, 110.0, 108.1, 74.7, 56.6, 56.5; Could not be observed by MS; HPLC Method B, Retention time ‐ 4.8 min, 88 %.

#### *tert*‐Butyl‐(*E*)‐3‐(2‐((*tert*‐butoxycarbonyl)(4‐(3‐(((4,5‐dimethoxy‐2‐nitrobenzyl)oxy)amino)‐3‐oxoprop‐1‐en‐1‐yl)benzyl)amino)ethyl)‐2‐methyl‐1*H*‐indole‐1‐carboxylate (10)

Working in the dark as far as possible, PyBOP (209 mg, 0.402 mmol, 1.1 eq) was added to a solution of (*E*)‐3‐(4‐((t*ert*‐butyloxycarbonyl‐(2‐(1‐(*tert*‐butyloxycarbonyl)‐2‐methyl‐1*H*‐indol‐3‐yl)ethyl)amino)methyl)phenyl)prop‐2‐enoic acid (**9**) (196 mg, 0.365 mmol, 1.0 eq) and triethylamine (152 μL, 1.10 mmol, 3.0 eq) in dry tetrahydrofuran (3.7 mL). The reaction mixture was stirred for 15 min at rt before *O*‐(4,5‐dimethoxy‐2‐nitrobenzyl)‐hydroxylamine (**8**) (100 mg, 0.439 mmol, 1.2 eq) was added. Stirring was continued at rt for 18 h, then the reaction mixture was diluted with ethyl acetate (20 mL) and quenched with aqueous 1 M solution of hydrochloric acid (10 mL). The reaction mixture was extracted with ethyl acetate (20 mL), the combined organic components were washed with a saturated solution of sodium hydrogen carbonate (40 mL), water (40 mL), and brine (40 mL) then dried (MgSO_4_), filtered, and concentrated *in vacuo*. Purification using column chromatography (elution with 0–1 % ethanol:chloroform) yielded the title compound (**10**) (201 mg, 74 %) as a yellow solid. *R_f_* 0.29 (50 % ethyl acetate:petroleum ether); mp 88–92 °C (from CHCl_3_); *ν*_max_ (thin film)/cm^−1^ 3192, 2977, 2932, 1728, 1686, 1521, 1460, 1366, 1323, 1276, 1220, 1159, 1137, 1117, 1068; ^1^H NMR(500 MHz, D_6_‐DMSO) δ_H_ 11.00 (1H, br s), 8.00 (1H, br d, *J* 8.1), 7.67 (1H, s), 7.50 (2H, d, *J* 8.0), 7.46 (1H, d, *J* 15.5), 7.42 (1H, br d, *J* 7.5), 7.42 (1H, s), 7.24 (2H, d, *J* 8.0), 7.21 (1H, ddd, *J* 8.1, 7.1, 1.3), 7.16 (1H, ddd, *J* 7.5, 7.1, 1.2), 6.46 (1H, d, *J* 15.5), 5.25 (2H, s), 4.41 (2H, s), 3.95 (3H, s), 3.33 (2H, t, *J* 7.2), 3.02 (2H, s), 2.83 (2H, t, *J* 7.2), 2.46 (3H, s), 1.64 (9H, s), 1.34 (9H, s); ^13^C NMR (126 MHz, (D_6_‐DMSO) δ_C_ 163.6*, 154.4, 153.0, 149.5, 147.9, 140.0, 139.2, 134.9, 133.2, 132.9, 129.1, 127.4, 127.2, 126.2, 122.7, 121.7, 117.8, 117.1, 114.8, 114.4, 112.1, 108.3, 83.1, 78.5, 73.2, 56.0, 55.9, 49.6, 46.1, 27.6, 27.5, 27.4, 21.4, 12.7; HRMS *m/z* (ESI^−^) Found: 743.33043, C_40_H_47_N_4_O_10_ requires [M−H]^−^ 743.32977; LRMS (ESI^−^) 743 ([M−H]^−^, 100 %); HPLC Method A, Retention time – 13.4 min, >99 %. *Peak at 163.6 ppm confirmed by comparison to spectra of related compounds.

#### (*E*)‐*N*‐(4‐(3‐((4,5‐Dimethoxy‐2‐nitrobenzyl)oxy)‐3‐oxoprop‐1‐en‐1‐yl)benzyl)‐2‐(2‐methyl‐1*H*‐indol‐3‐yl)ethan‐1‐aminium trifluoroacetate (1, Zap‐Pano⋅TFA)

Trifluoroacetic acid (2.7 mL, 20 % *v/v*) was added dropwise to a rapidly stirred solution of *tert*‐butyl‐(*E*)‐3‐(2‐((*tert*‐butoxycarbonyl)(4‐(3‐(((4,5‐dimethoxy‐2‐nitrobenzyl)oxy)amino)‐3‐oxoprop‐1‐en‐1‐yl)benzyl)amino)ethyl)‐2‐methyl‐1*H*‐indole‐1‐carboxylate (**10**) (0.10 g, 0.13 mmol, 1.0 eq) and triisopropylsilane (5.9 μL, 0.027 mmol, 0.2 eq) in dichloromethane (13 mL). The reaction mixture was stirred for 75 mins, until TLC analysis showed complete consumption of starting material. The reaction mixture was then diluted with toluene (1 mL) and dried by azeotrope with toluene (3×1 mL) *in vacuo*. Purification using column chromatography (elution with 1 : 3 : 40 water: isopropanol: ethyl acetate) yielded the title compound (**1**) (46 mg, 51 %) as a yellow solid. *R_f_* 0.26 (1 : 2 : 10 water: isopropanol: ethyl acetate); mp 106–110 °C (from ethanol); *ν*_max_ (thin film)/cm^−1^ 2967, 2849, 1669, 1626, 1521, 1461, 1331, 1277, 1201, 1136, 1068; ^1^H NMR (500 MHz, CD_3_OD) δ_H_ 7.68 (1H, s), 7.59 (2H, d, *J* 8.2), 7.59 (1H, d, *J* 15.8), 7.46 (2H, d, *J* 8.2), 7.39 (1H, br d, *J* 7.8), 7.39 (1H, s), 7.24 (1H, br d, *J* 8.0), 7.02 (ddd, *J* 8.0, 7.1, 1.0), 6.95 (1H, ddd, *J* 7.8, 7.1, 1.0), 6.48 (1H, d, *J* 15.8), 5.31 (2H, s), 4.18 (2H, s), 3.96 (3H, s), 3.88 (3H, s), 3.19–3.16 (2H, m), 3.09–3.06 (2H, m), 2.37 (3H, s); ^13^C NMR (126 MHz, CD_3_OD) δ_C_ 165.8, 155.0, 149.9, 141.6, 141.4, 137.2, 137.0, 135.1, 133.9, 131.3, 129.5, 129.3, 128.0, 121.8, 119.9, 119.3, 118.0, 112.5, 111.6, 109.2, 106.0, 75.5, 57.0, 56.8, 52.0, 49.0,* 22.5, 11.3; HRMS *m/z* (ESI^+^) Found: 545.23854, C_30_H_33_N_4_O_6_ requires [M+H]^+^ 545.23946; LRMS (ESI^+^) 567 ([M+Na]^+^, 100 %), 545 ([M+H]^+^,75 %), 528 (53 %), 384 (47 %), 371 (35 %), 174 (27 %), 129 (47); HPLC Method B, Retention time – 7.2 min, 96–98 % at 3 wavelengths, mean purity 97 %. All batches used for biological testing were >95 % purity. *Signal at 49.0 ppm overlaps with solvent peaks, see SI for expansion.

#### (*E*)‐3‐(4‐((t*ert*‐Butyloxycarbonyl‐(2‐(1‐(*tert*‐butyloxycarbonyl)‐2‐methyl‐*1H*‐indol‐3‐yl)ethyl)amino)methyl)phenyl)prop‐2‐enamide (S1)

PyBOP (32 mg, 0.062 mmol, 1.1 eq) was added to a solution of (*E*)‐3‐(4‐([t*ert*‐butyloxycarbonyl‐(2‐(1‐(*tert*‐butyloxycarbonyl)‐2‐methyl‐*1H*‐indol‐3‐yl)ethyl)amino)methyl)phenyl)prop‐2‐enoic acid (**9**) (30 mg, 0.056 mmol, 1.0 eq) and triethylamine (0.12 mL, 0.84 mmol, 15 eq) in dry tetrahydrofuran (0.6 mL). The reaction mixture was stirred for 15 min at rt before ammonium chloride (30 mg, 0.56 mmol, 10 eq) was added. Stirring was continued at rt for 18 h, then the reaction mixture was diluted with ethyl acetate (20 mL) and quenched with aqueous 1 M solution of hydrochloric acid (10 mL). The reaction mixture was extracted with ethyl acetate (20 mL), the organic components were washed with a saturated solution of sodium hydrogen carbonate (40 mL), water (40 mL), and brine (40 mL), then dried (MgSO_4_), filtered, and concentrated *in vacuo*. Purification using column chromatography (elution with 80 % ethyl acetate:petroleum ether) yielded the title compound (**S1**) (25 mg, 84 %) as a colourless solid. *R_f_* 0.55 (100 % ethyl acetate); mp 97–100 °C (from CHCl_3_); *ν*_max_ (thin film)/cm^−1^ 3340, 3189, 2976, 2930, 1729, 1670, 1607, 1460, 1393, 1323, 1253, 1159, 1137, 1117; ^1^H NMR (500 MHz, D_6_‐DMSO) δ_H_ 8.01 (ddd, *J*=8.3, 1.3, 0.6 Hz, 1H), 7.49 (d, *J* 8.1, 2H), 7.41 (d, *J* 7.1, 1H), 7.41 (d, *J* 15.9, 1H), 7.24 (d, *J* 8.1, 2H), 7.21 (ddd, *J* 8.3, 7.3, 1.5, 1H), 7.17 (ddd, *J* 7.3, 7.1, 1.3, 1H), 6.58 (d, *J* 15.9, 1H), 4.41 (s, 2H), 3.33 (dd, *J* 8.1, 6.4, 2H), 2.83 (dd, *J* 8.1, 6.4, 2H), 2.46 (s, 3H), 1.64 (s, 9H), 1.34 (s, 9H); ^13^C NMR at (126 MHz, D_6_‐DMSO) δ_C_ 166.3, 154.5, 149.6, 139.6, 138.3, 135.0, 133.6, 132.9, 129.1, 127.5, 127.1, 122.7, 122.0, 121.8, 117.2, 114.9, 114.4, 83.2, 78.5, 49.6, 46.0, 27.5, 27.4, 22.1, 12.7; HRMS *m/z* (ESI^+^) Found: 556.2780, C_31_H_39_N_3_O_5_Na requires [M+Na]^+^ 556.2782; LRMS (ESI^+^) 556 ([M+Na]^+^, 83 %), 534 ([M+H]^+^, 24 %), 478 (27 %), 378 (15 %), 361 (42 %), 317 (100 %), 300 (56 %); HPLC Method A, Retention time – 11.5 min, >99 %.

#### (*E*)‐3‐(4‐(((2‐(2‐Methyl‐1*H*‐indol‐3‐yl)ethyl)amino)methyl)phenyl)prop‐2‐enamide (15)

Trifluoroacetic acid (0.20 mL, 20 % *v/v*) was added dropwise to a rapidly stirred solution of (*E*)‐3‐(4‐((t*ert*‐butyloxycarbonyl‐(2‐(1‐(*tert*‐butyloxycarbonyl)‐2‐methyl‐1*H*‐indol‐3‐yl)ethyl)amino)methyl)phenyl)prop‐2‐enamide (**S1**) (15 mg, 0.020 mmol, 1.0 eq) and triisopropylsilane (0.83 μL, 4.0 μmol, 0.2 eq) in dichloromethane (1 mL). The reaction mixture was stirred for 60 min, until TLC analysis indicated complete consumption of starting material, then diluted with toluene (1 mL) and dried by azeotroping with toluene (3×1 mL) *in vacuo*. Purification using column chromatography (elution with 1 : 3 : 40 H_2_O: isopropyl alcohol: ethyl acetate) yielded the title compound (**15**) (7.1 mg, 65 %) as a pale‐yellow solid. *R_f_* 0.26 (1 : 2 : 10 H_2_O: isopropyl alcohol: ethyl acetate); mp 82–88 °C (from CHCl_3_); *ν*_max_ (thin film)/cm^−1^ 3394, 3196, 2922, 2852, 1665, 1628, 1593, 1461, 1425,1397, 1202, 1181, 1135; ^1^H NMR (400 MHz, CD_3_OD) δ_H_ 7.53 (1H, d, *J* 15.7), 7.50 (2H, d, *J* 8.1), 7.38 (1H, ddd, *J* 7.9, 1.1, 1.0), 7.28 (2H, d, *J* 8.1), 7.23 (1H, ddd, *J* 8.1, 1.1, 1.0), 6.99 (1H, ddd, *J* 8.1, 7.1, 1.1), 6.92 (1H, ddd, *J* 7.9, 7.1, 1.1), 6.62 (1H, d, *J* 15.7), 3.80 (2H, s), 2.98–2.90 (2H, m), 2.89–2.80 (2H, m), 2.34 (3H, s); ^13^C NMR (126 MHz, CD_3_OD) δ_C_ 170.9, 142.2, 140.5, 137.1, 135.6, 133.4, 130.3, 129.7, 129.2, 121.5, 121.5, 119.6, 118.3, 111.4, 108.0, 53.3, 49.9, 24.2, 11.4; HRMS *m/z* (ESI^+^) Found: 334.19135, C_21_H_24_N_3_O requires [M+H]^+^ 334.19139; LRMS (ESI^+^) 334 ([M+H]^+^, 100 %), 158 (18 %);HPLC Method B, Retention time – 6.6 min, 91–96 % at 3 wavelengths, mean purity 94 %.

## Conflict of interest

The authors declare no conflict of interest.

## Supporting information

As a service to our authors and readers, this journal provides supporting information supplied by the authors. Such materials are peer reviewed and may be re‐organized for online delivery, but are not copy‐edited or typeset. Technical support issues arising from supporting information (other than missing files) should be addressed to the authors.

Supporting Information
