# Supplementary material for: Zap‐Pano: a Photocaged Prodrug of the KDAC Inhibitor Panobinostat
Source: ChemMedChem. 2021 Jul 29;16(24):3691–700. doi: 10.1002/cmdc.202100403 (PMC9291796; doi:10.1002/cmdc.202100403)
Supplement: Supplementary file 1 — Supporting Information [file CMDC-16-3691-s001.pdf]

ChemMedChem

Supporting Information

Zap-Pano: a Photocaged Prodrug of the KDAC Inhibitor Panobinostat

Kathrin S. Troelsen, Ewen D. D. Calder+, Anna Skwarska+, Deborah Sneddon, Ester M. Hammond,* and Stuart J. Conway*

Table of contents

NMR spectra of novel and biologically tested compounds S2

HPLC traces of novel and biologically tested compounds S14

LCMS traces comparing the Pano-NH_2 and Pano acid S21

N-Phthalimido-*O*-(4, 5-dimethoxy-2-nitrobenzyl)-hydroxylamine (**7**)

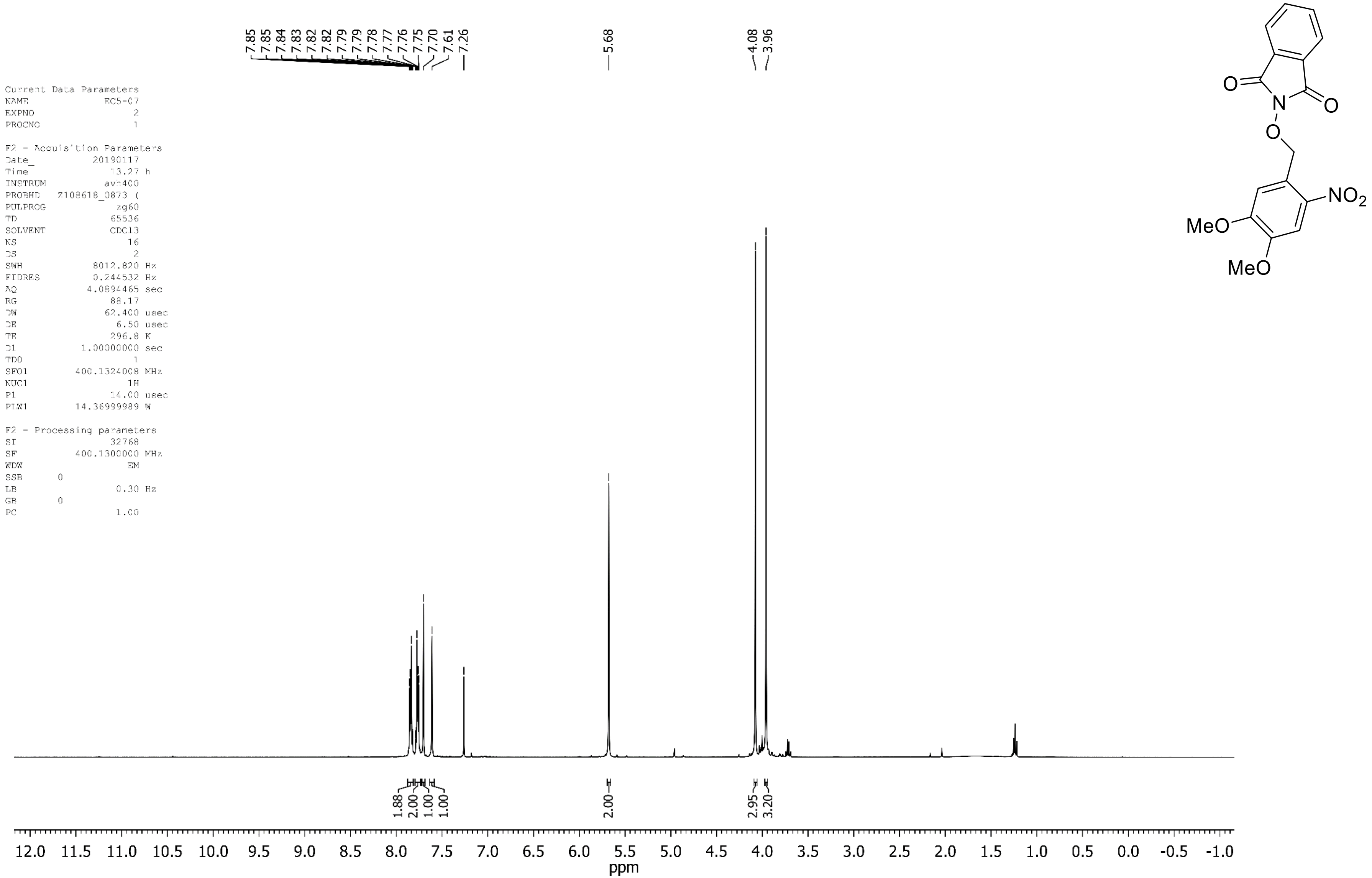

N-Phthalimido-*O*-(4, 5-dimethoxy-2-nitrobenzyl)-hydroxylamine (**7**)

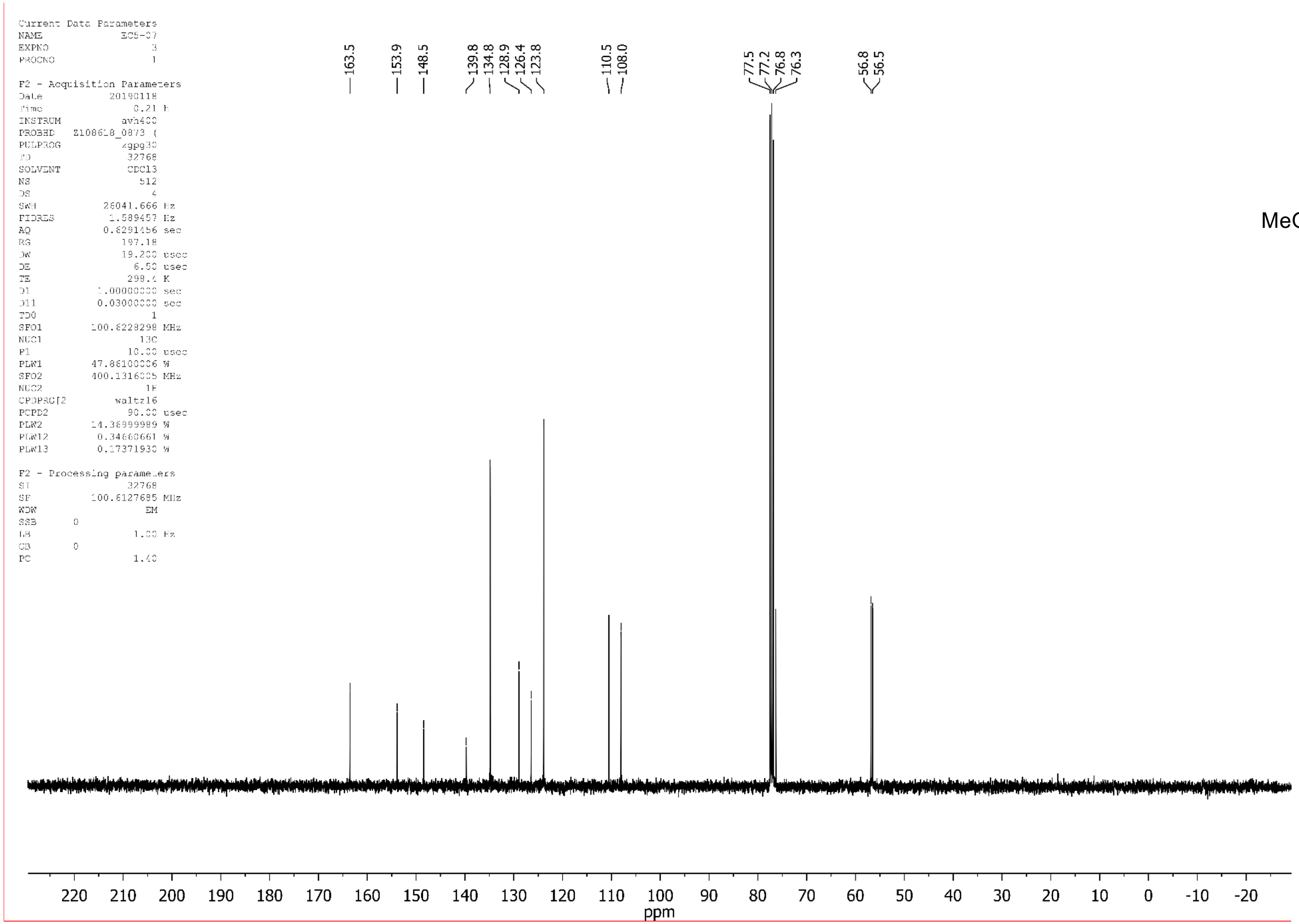

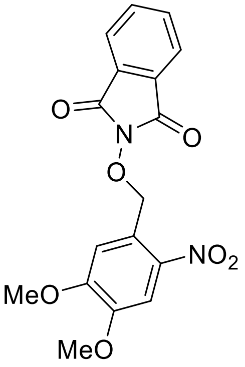

O-(4, 5-Dimethoxy-2-nitrobenzyl)-hydroxylamine (**8**)

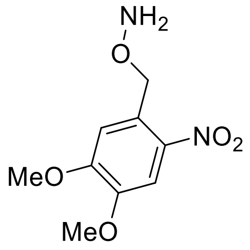

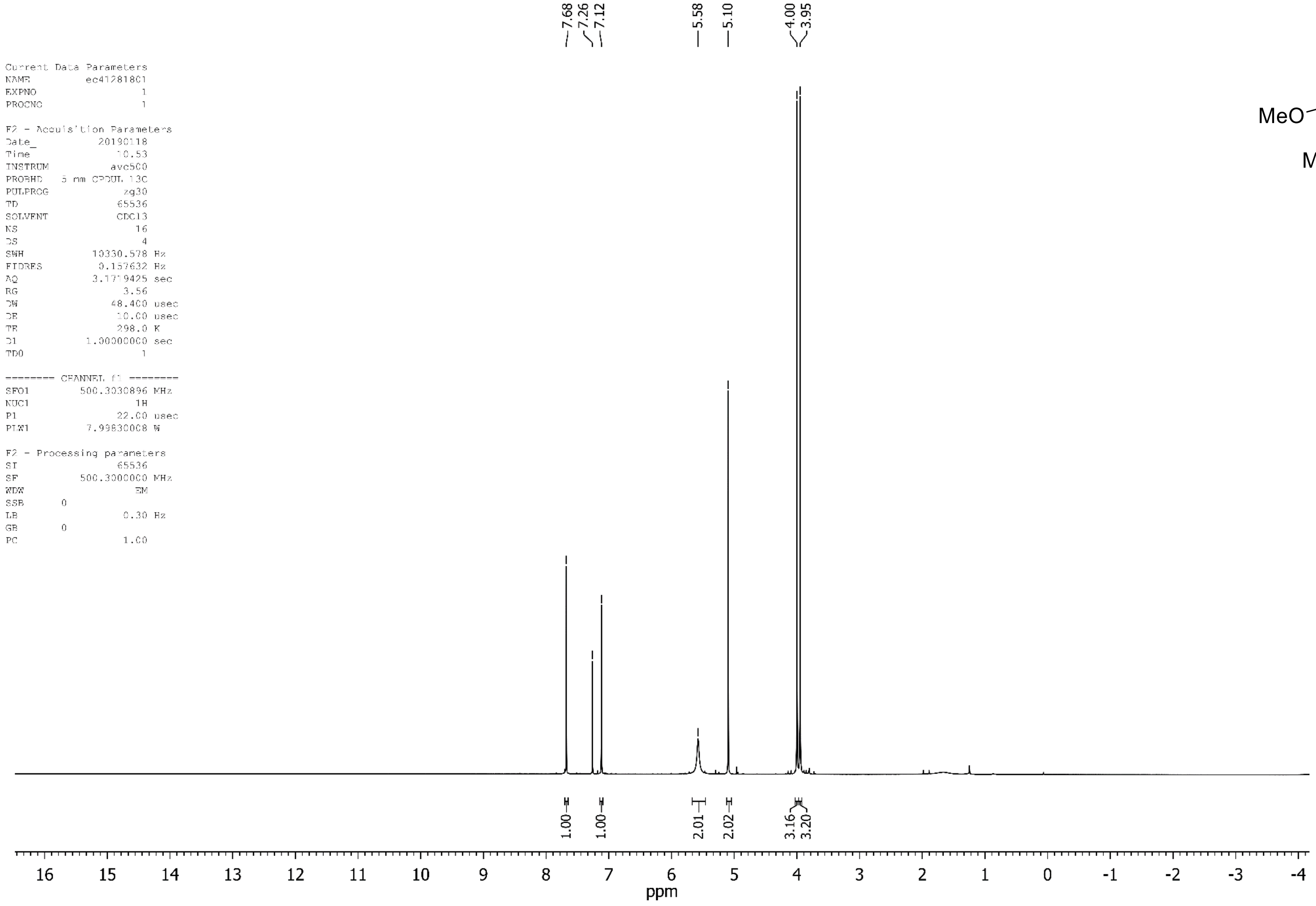

O-(4, 5-Dimethoxy-2-nitrobenzyl)-hydroxylamine (**8**)

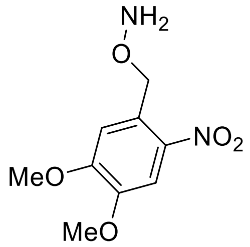

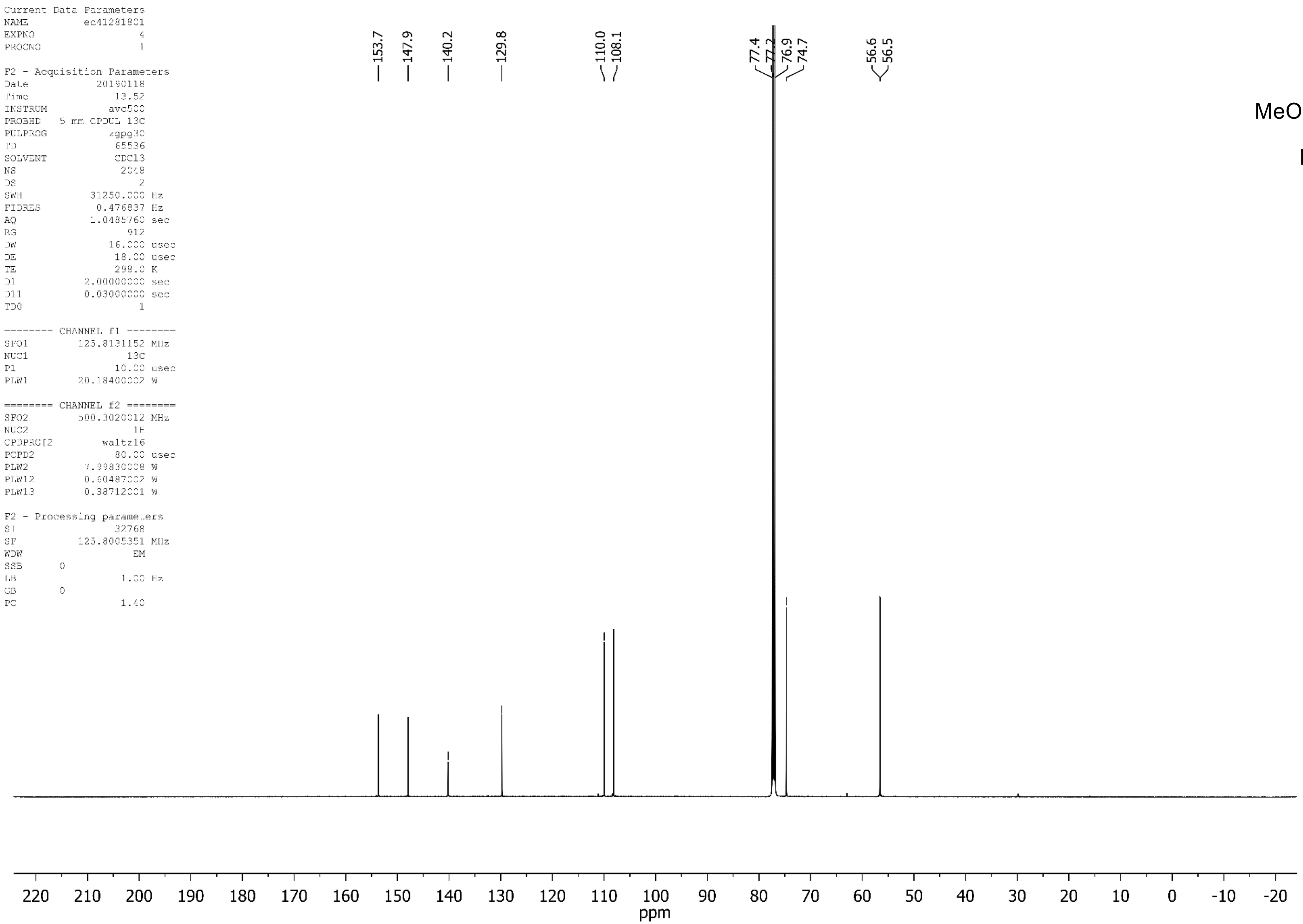

tert-Butyl-(*E*)-3-(2-((*tert*-butoxycarbonyl)(4-(3-(((4,5-dimethoxy-2-nitrobenzyl)oxy)amino)-3-oxoprop-1-en-1-yl)benzyl)amino)ethyl)-2-methyl-1*H*-indole-1-carboxylate (**10**)

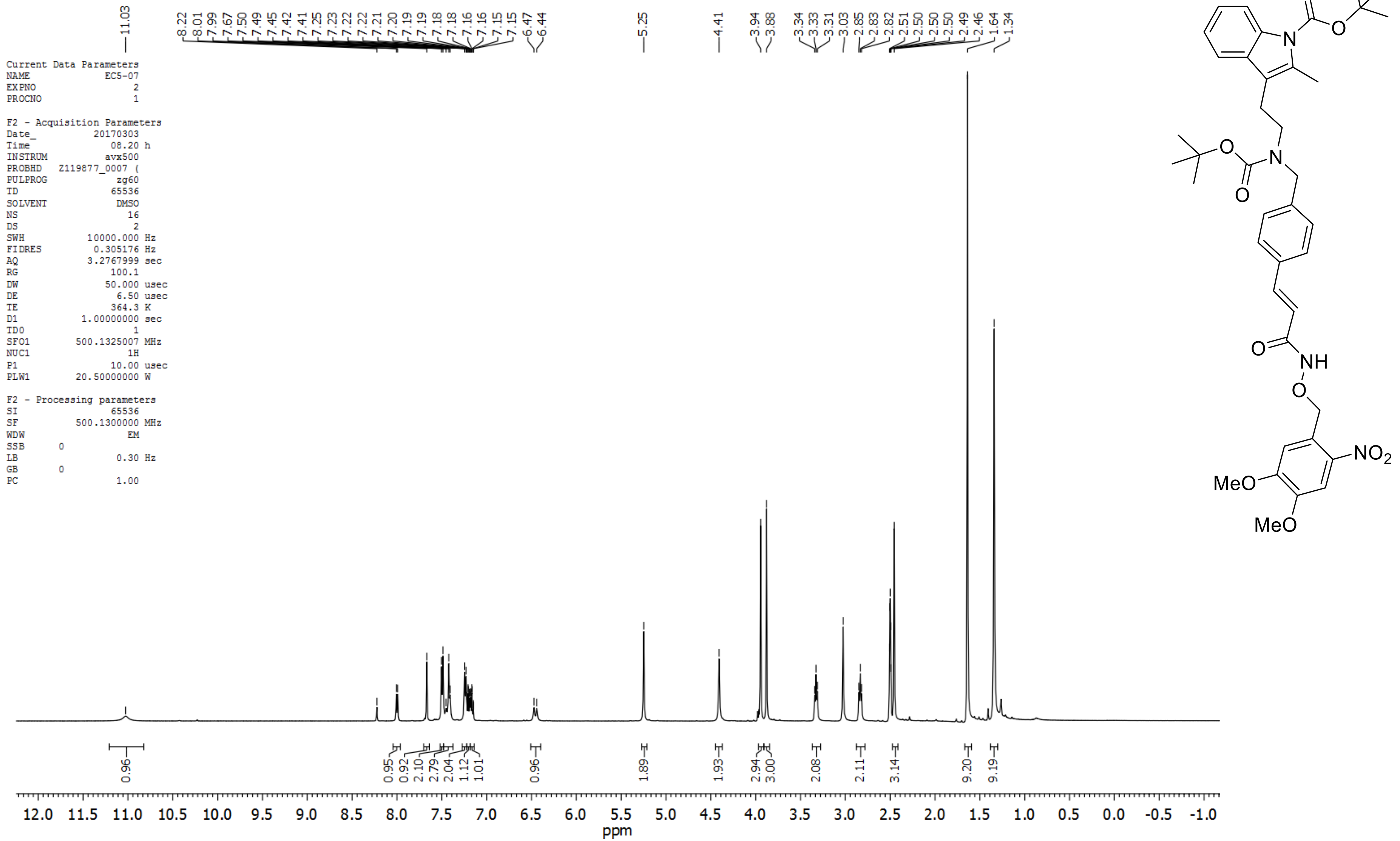

tert-Butyl-(*E*)-3-(2-((*tert*-butoxycarbonyl)(4-(3-(((4,5-dimethoxy-2-nitrobenzyl)oxy)amino)-3-oxoprop-1-en-1-yl)benzyl)amino)ethyl)-2-methyl-1*H*-indole-1-carboxylate (**10**)

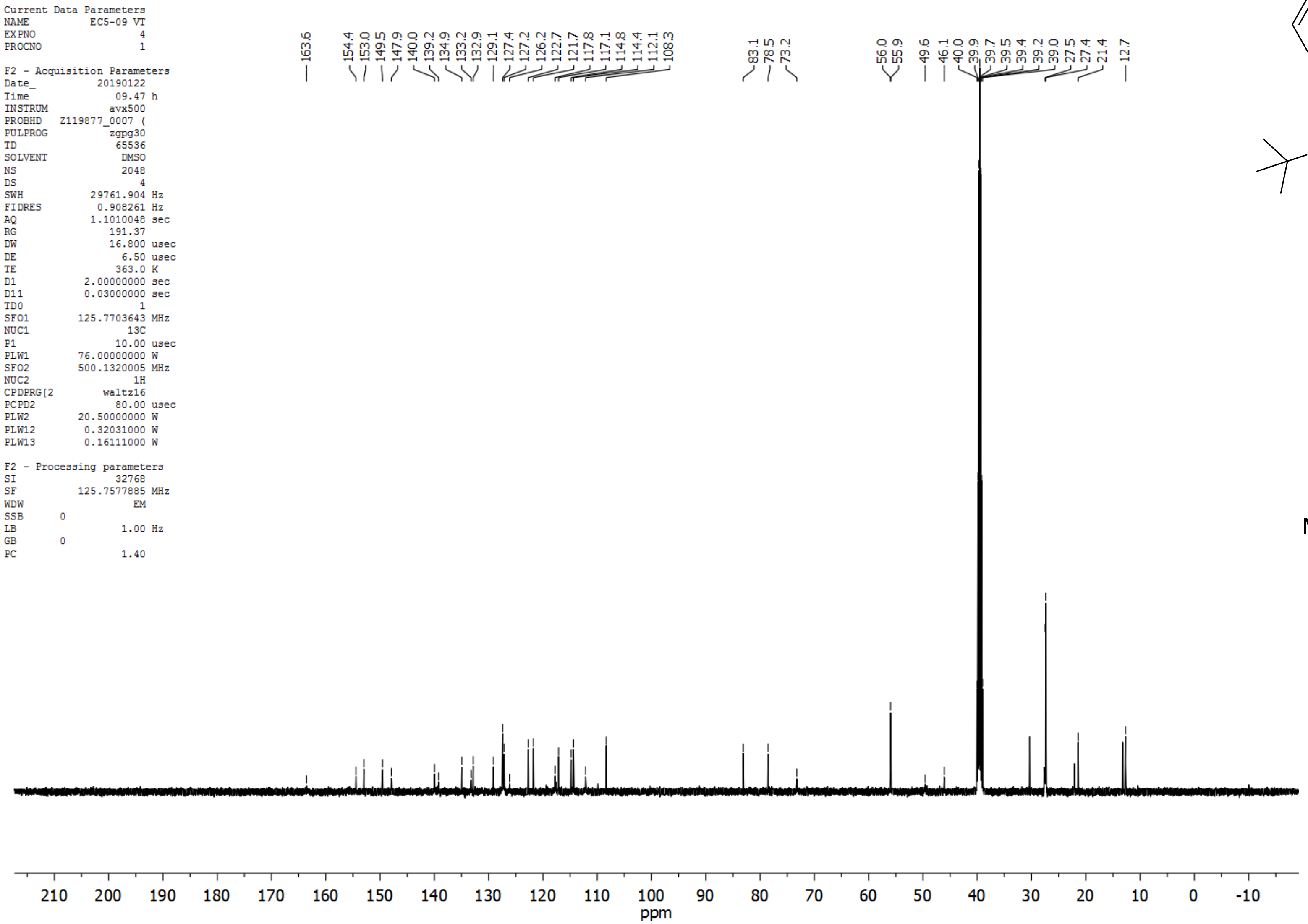

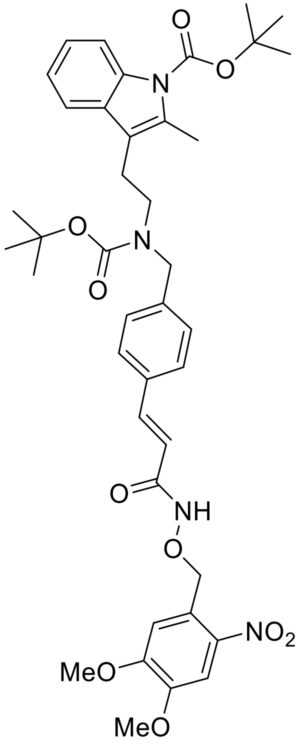

(*E*)-*N*-(4-(3-((4,5-Dimethoxy-2-nitrobenzyl)oxy)-3-oxoprop-1-en-1-yl)benzyl)-2-(2-methyl-1*H*-indol-3-yl)ethan-1-aminium trifluoroacetate (**1**, Zap-Pano·TFA)

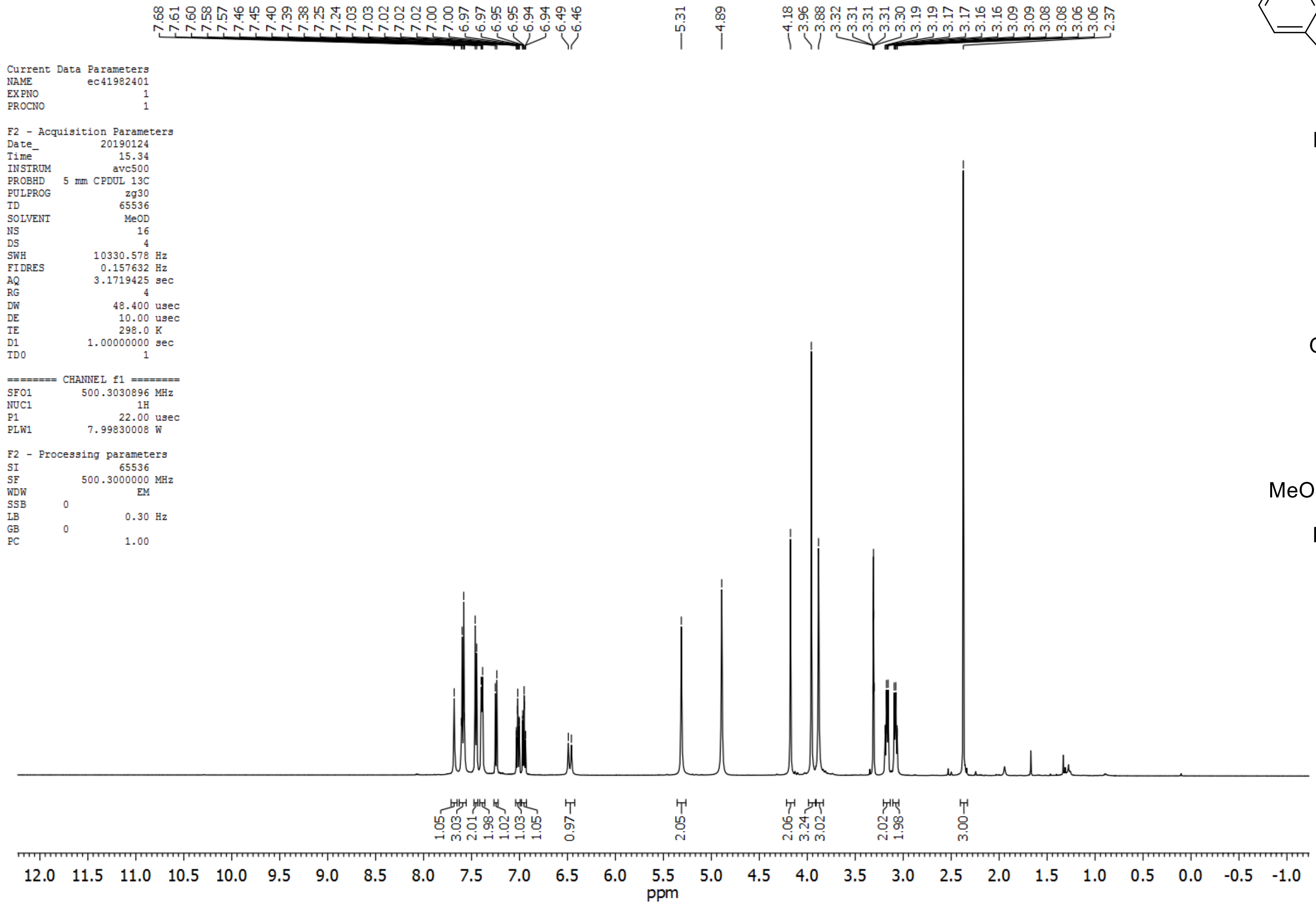

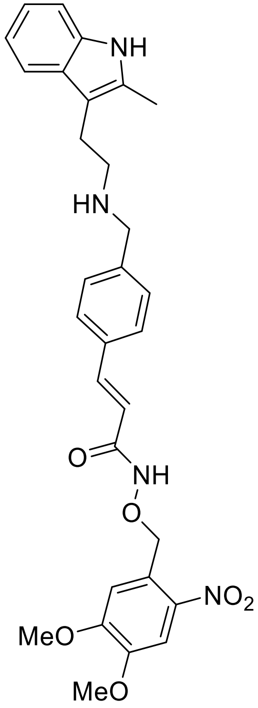

(*E*)-*N*-(4-(3-((4,5-Dimethoxy-2-nitrobenzyl)oxy)-3-oxoprop-1-en-1-yl)benzyl)-2-(2-methyl-1*H*-indol-3-yl)ethan-1-aminium trifluoroacetate (**1**, Zap-Pano·TFA)

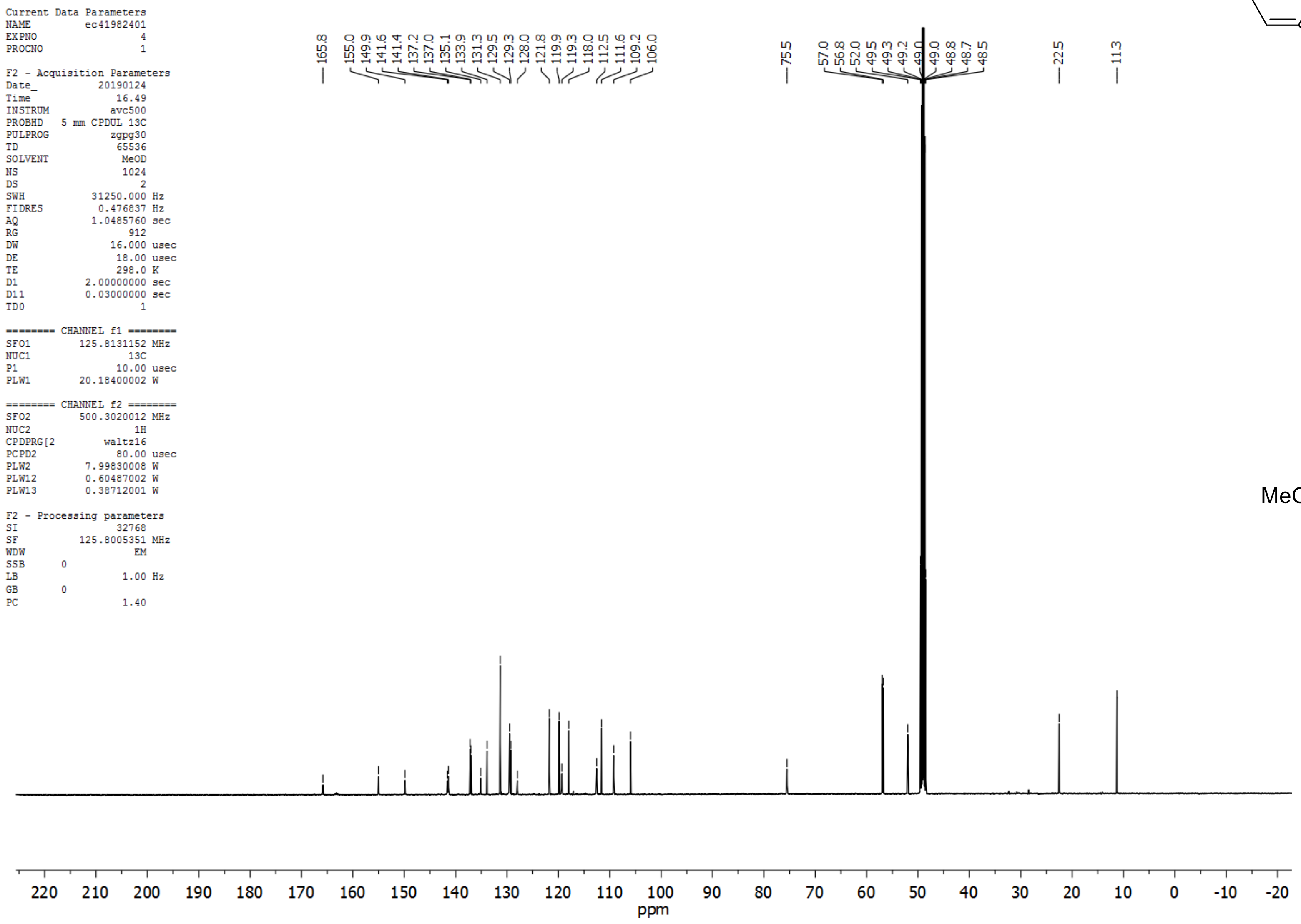

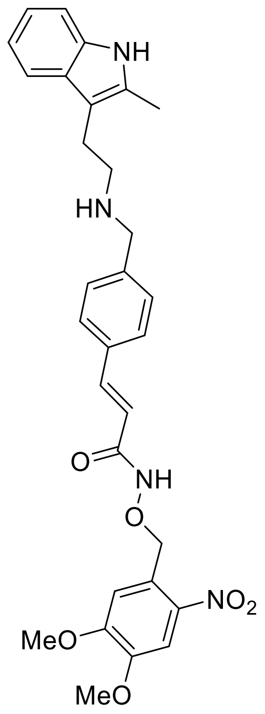

(*E*)-3-(4-{[*tert*-Butyloxycarbonyl-(2-{1-[*tert*-butyloxycarbonyl]-2-methyl-*1H*-indol-3-yl}ethyl)amino]methyl}phenyl)prop-2-enamide (**S1**)

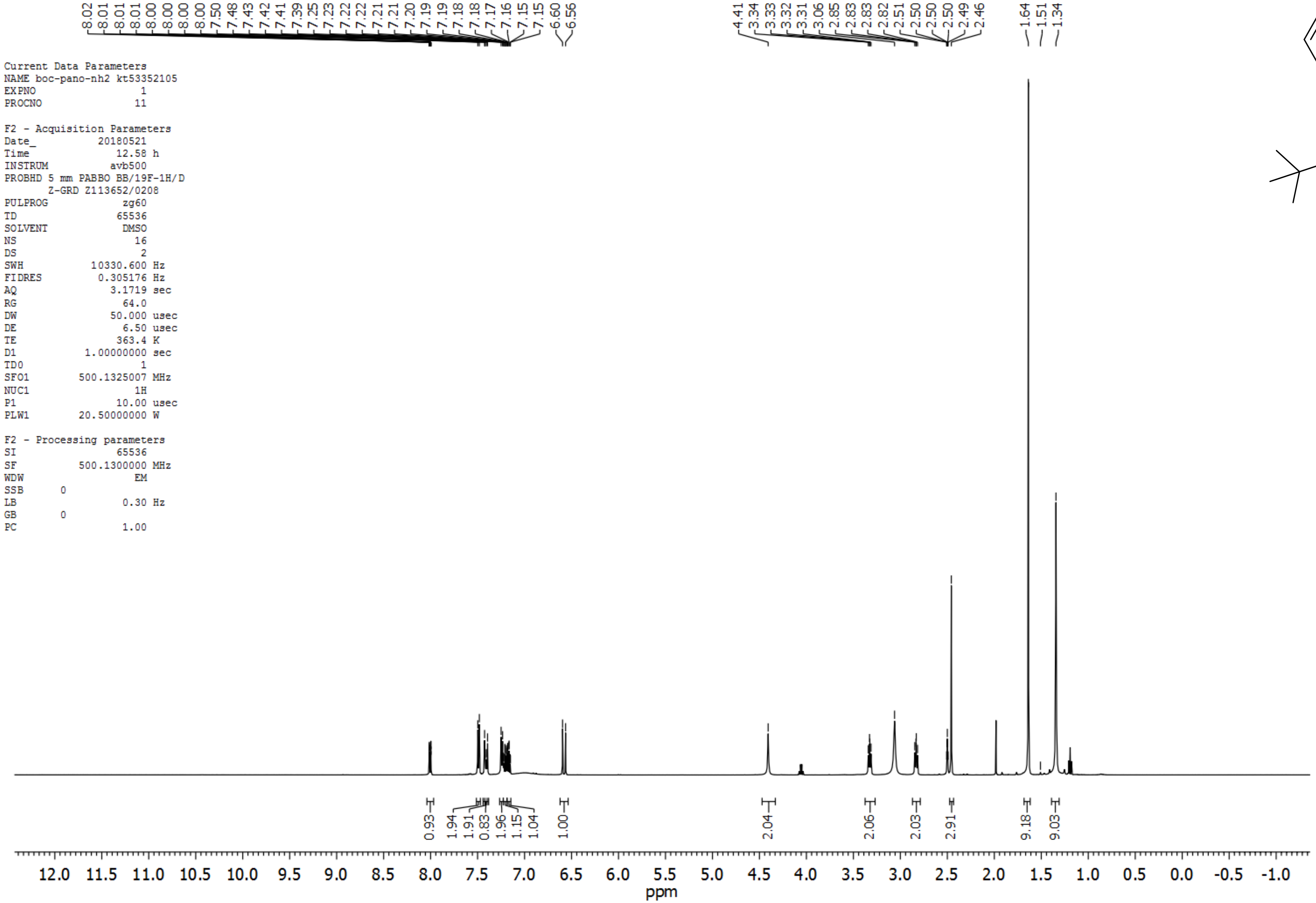

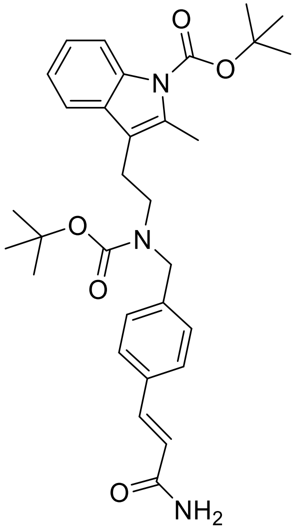

(*E*)-3-(4-{[*tert*-Butyloxycarbonyl-(2-{1-[*tert*-butyloxycarbonyl]-2-methyl-*1H*-indol-3-yl}ethyl)amino]methyl}phenyl)prop-2-enamide (**S1**)

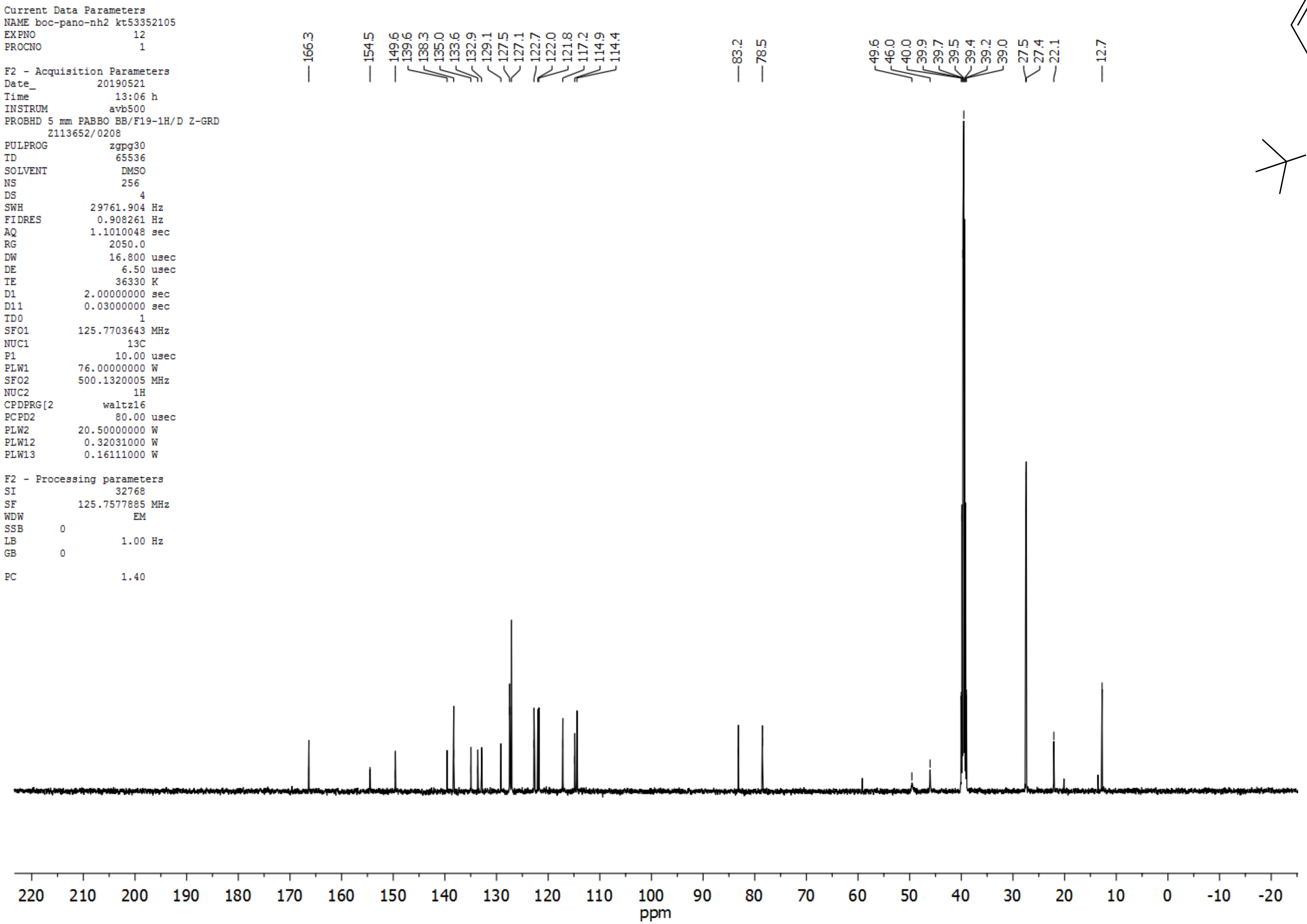

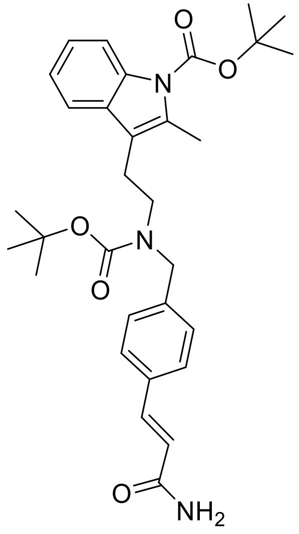

(*E*)-3-(4-{[(2-{2-Methyl-*1H*-indol-3-yl}ethyl)amino]methyl}phenyl)prop-2-enamide trifluoroacetate (**15**)

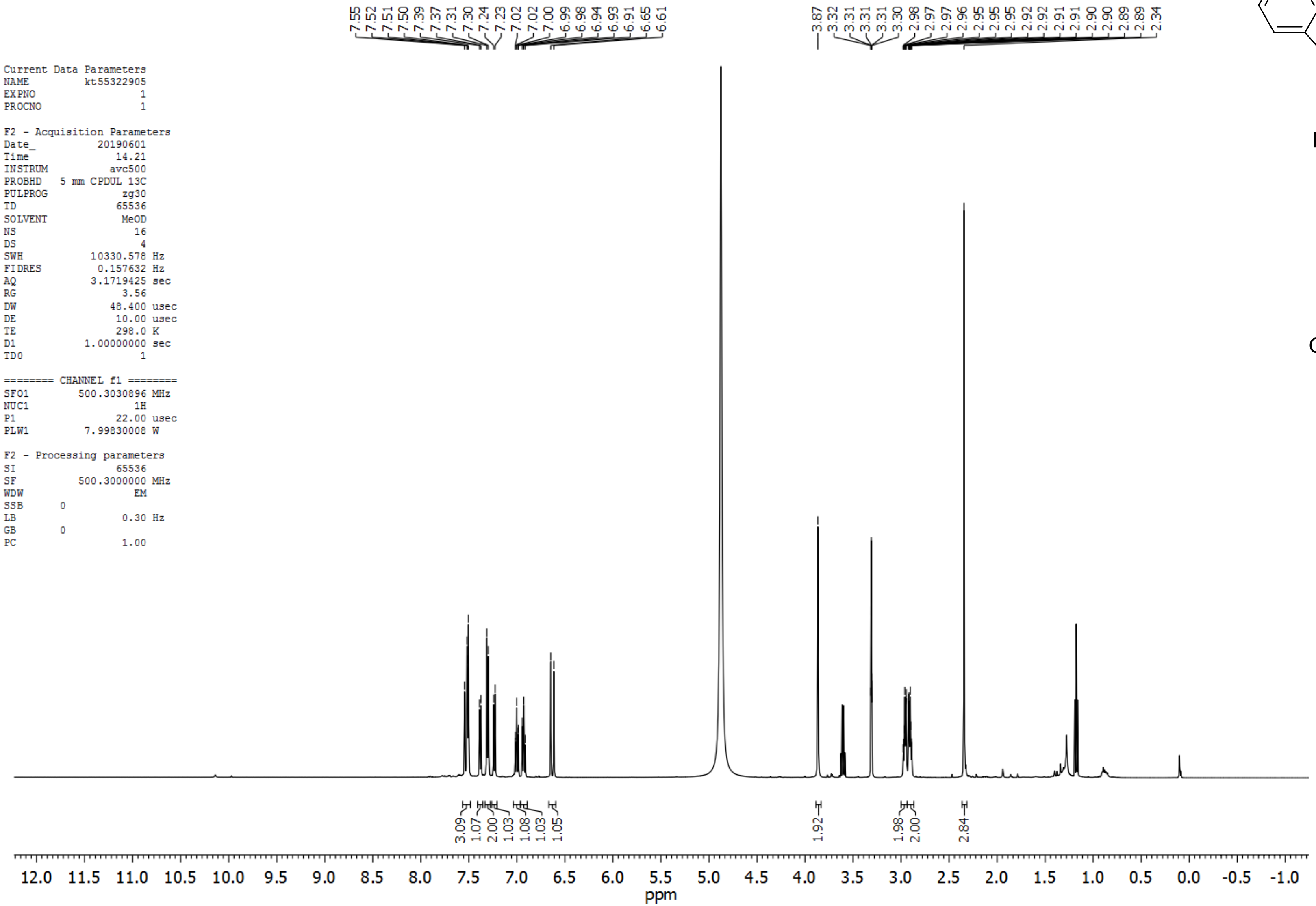

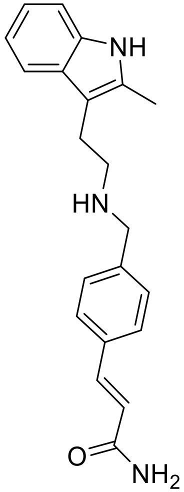

(*E*)-3-(4-{[(2-{2-Methyl-*1H*-indol-3-yl}ethyl)amino]methyl}phenyl)prop-2-enamide trifluoroacetate (**15**)

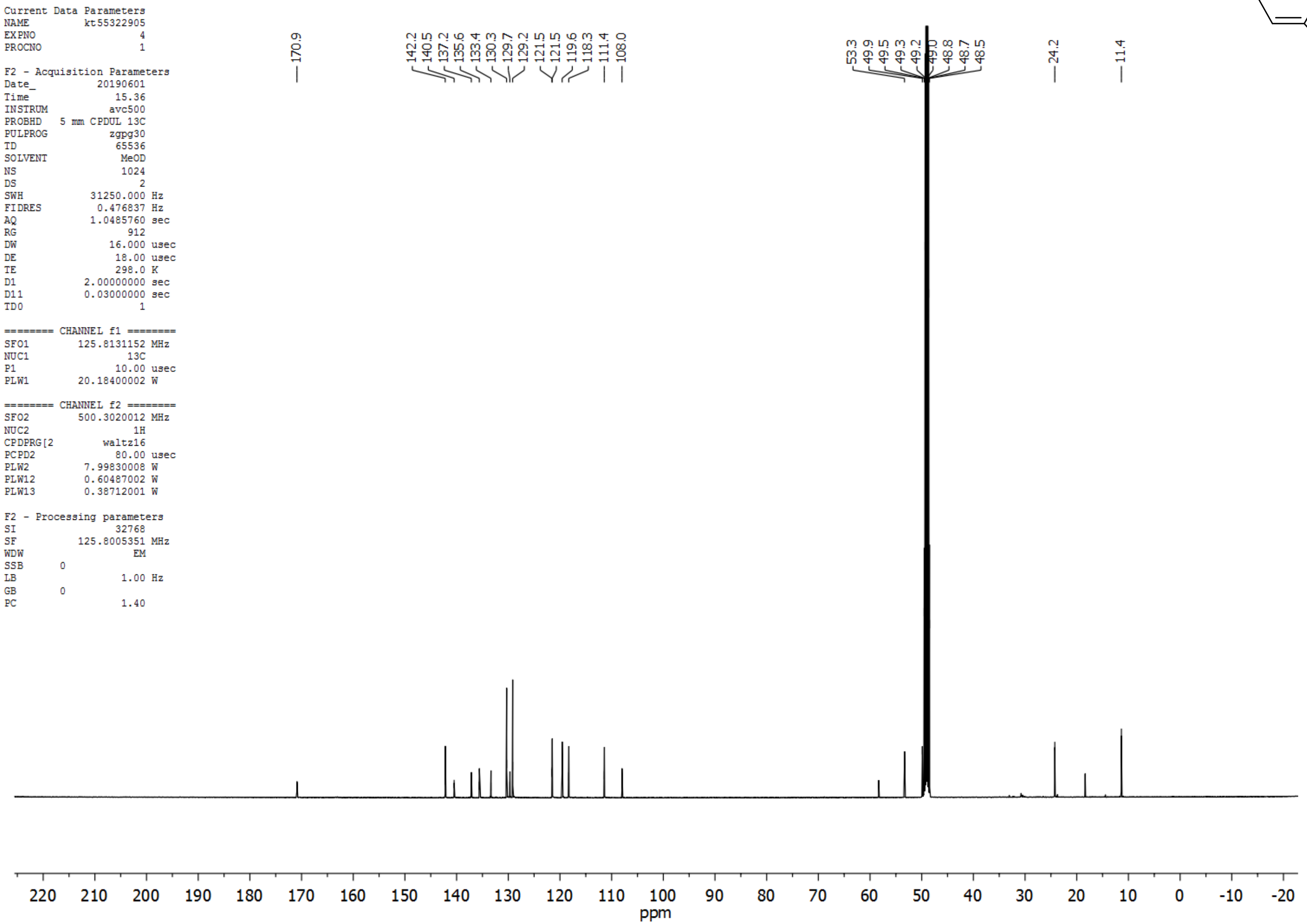

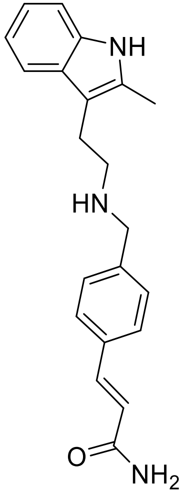

Panobinostat (**2**)

EC4-14 Pano 2/26/2018

Acquisition Method	Purity short run @254 nm
Acquisition Date/Time	2/26/2018 6:56 pm
Injection Volume	10
Sample Name	EC4-14 Pano
Sample Description	

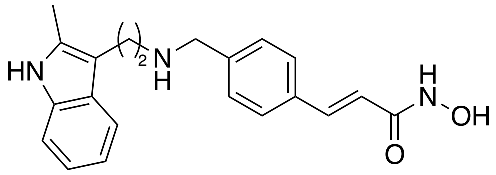

EC4-14 Pano : Injection 1

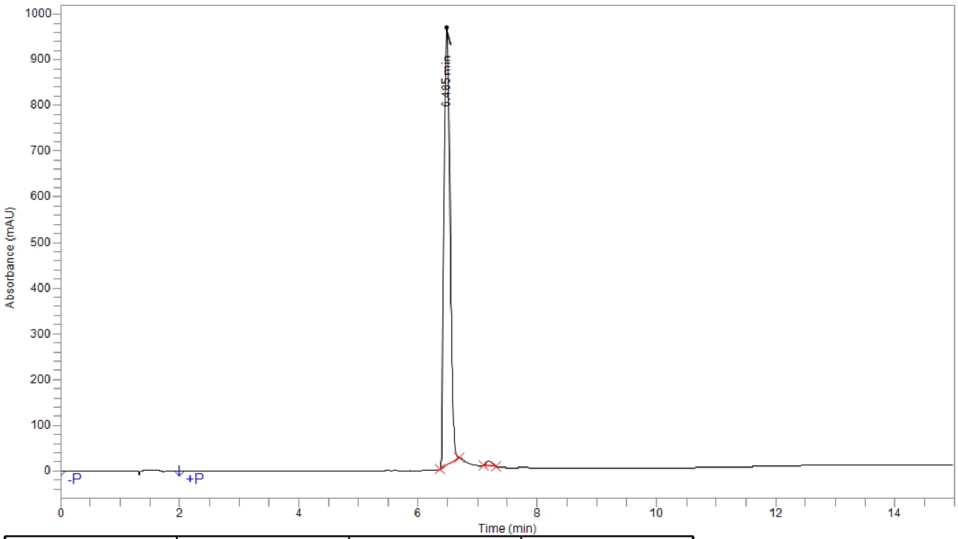

Time	Height	Area	Area %
6.485	958,967.0	6,619,001.0	98.81
7.183	11,494.8	79,439.7	1.19
**Total**		6,698,440.7	100.00

(*E*)-*N*-(4-(3-((Benzyloxy)amino)-3-oxoprop-1-en-1-yl)benzyl)-2-(2-methyl-1*H*-indol-3-yl)ethan-1-aminium 2,2,2-trifluoroacetate (**14**)

EC5-03 **11/15/2018**

Acquisition Method	Purity short run @254 nm
Acquisition Date/Time	11/15/2018 10:41 am
Injection Volume	10
Sample Name	EC5-03
Sample Description	
Batch Description	

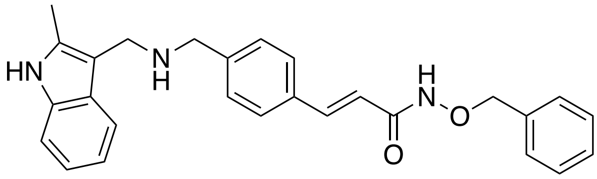

EC5-03 : Injection 1

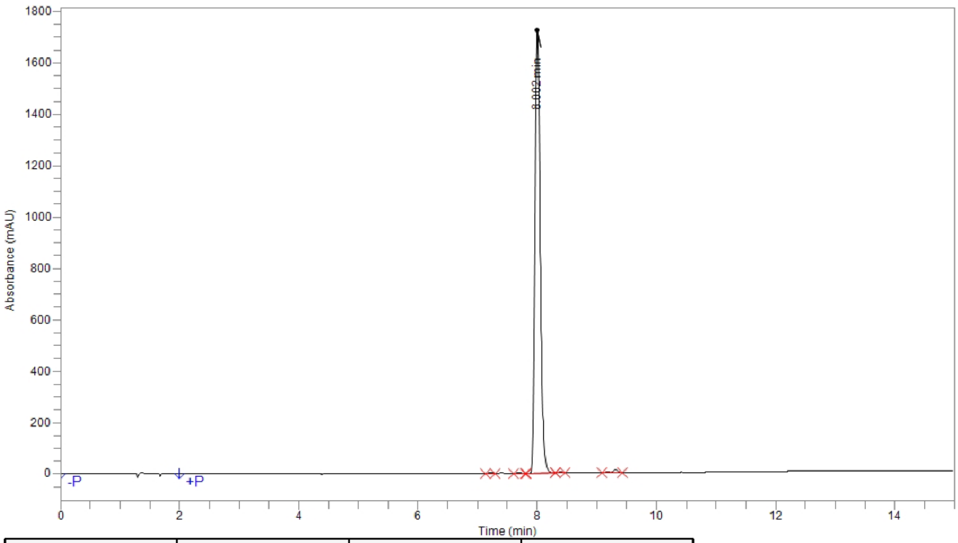

Time	Height	Area	Area %
7.223	3,653.0	16,559.7	0.16
7.696	3,057.3	14,995.5	0.14
8.002	1,727,141.3	10,237,501.0	98.80
8.393	4,153.3	17,709.5	0.17
9.185	3,472.0	16,907.9	0.16
9.317	11,731.4	58,166.0	0.56
**Total**		10,361,839.6	100.00

N-Phthalimido-*O*-(4, 5-dimethoxy-2-nitrobenzyl)-hydroxylamine (**7**)

EC5-07 2/4/2019

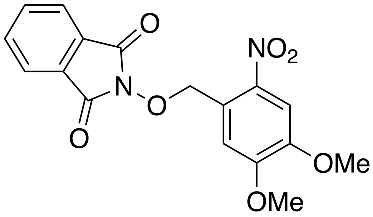

Acquisition Method	Purity short run @254 nm
Acquisition Date/Time	2/4/2019 2:56 pm
Injection Volume	15
Sample Name	EC5-07
Sample Description	
Batch Description	For start up

EC5-07 : Injection 1

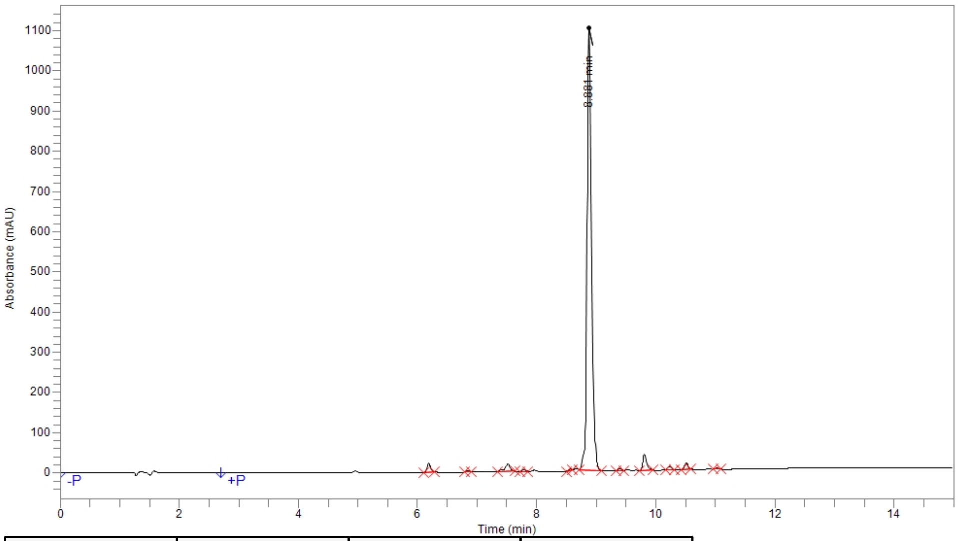

Time	Height	Area	Area %
6.187	22,291.7	95,260.7	1.56
6.849	3,125.7	10,874.1	0.18
7.515	19,069.4	121,228.5	1.98
7.786	4,729.3	18,356.1	0.30
8.568	3,096.0	9,847.7	0.16
8.881	1,100,867.4	5,542,449.0	90.73
9.396	5,132.9	19,542.5	0.32
9.812	40,597.3	180,004.5	2.95
10.230	7,652.0	28,388.1	0.46
10.512	16,526.1	67,792.6	1.11
11.025	3,921.4	14,825.9	0.24
**Total**		6,108,569.8	100.00

O-(4, 5-Dimethoxy-2-nitrobenzyl)-hydroxylamine (**8**)

Acquisition Method	Purity short run @254 nm
Acquisition Date/Time	2/4/2019 3:31 pm
Injection Volume	15
Sample Name	EC5-08
Sample Description	
Batch Description	For start up

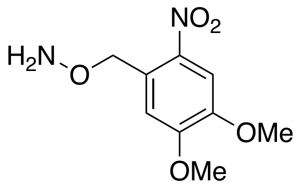

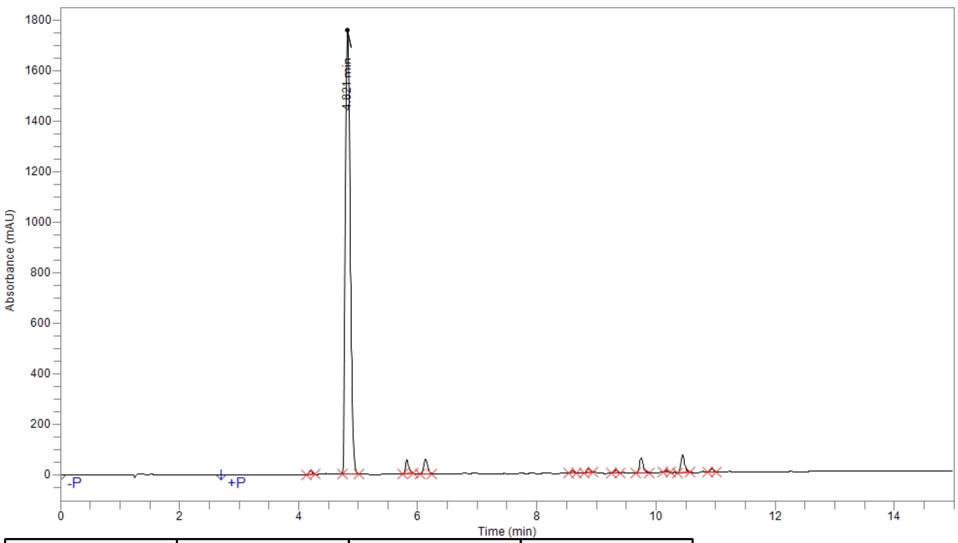

Time	Height	Area	Area %
4.203	14,582.9	56,795.7	0.51
4.821	1,758,260.8	9,880,647.6	88.18
5.818	55,634.5	228,306.6	2.04
6.129	57,109.0	245,435.0	2.19
8.598	9,865.6	41,665.7	0.37
8.868	17,319.8	69,371.1	0.62
9.326	10,320.8	40,380.4	0.36
9.750	59,452.5	268,264.5	2.39
10.176	7,656.6	26,268.1	0.23
10.446	69,466.2	294,405.6	2.63
10.940	13,207.9	53,766.9	0.48
**Total**		11,205,307.1	100.00

tert-Butyl-(*E*)-3-(2-((*tert*-butoxycarbonyl)(4-(3-(((4,5-dimethoxy-2-nitrobenzyl)oxy)amino)-3-oxoprop-1-en-1-yl)benzyl)amino)ethyl)-2-methyl-1*H*-indole-1-carboxylate (**10**)

EC2-46 **Purity short run @254 nm** **2/26/2017 4:01 pm**

Sample Name EC2-46
Vial Number 1
Injection Volume 10
Acquisition Date/Time 2/26/2017 3:42 pm
Acquisition Method Purity short run @254 nm
Processing Method Purity short run @254 nm

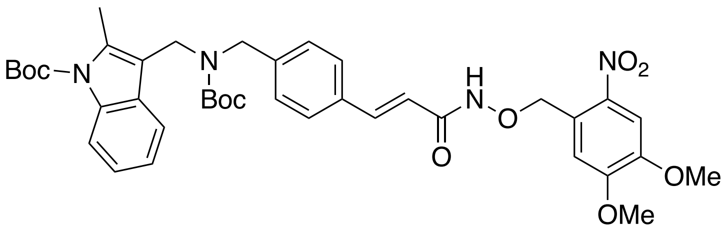

EC2-46 : Injection 1

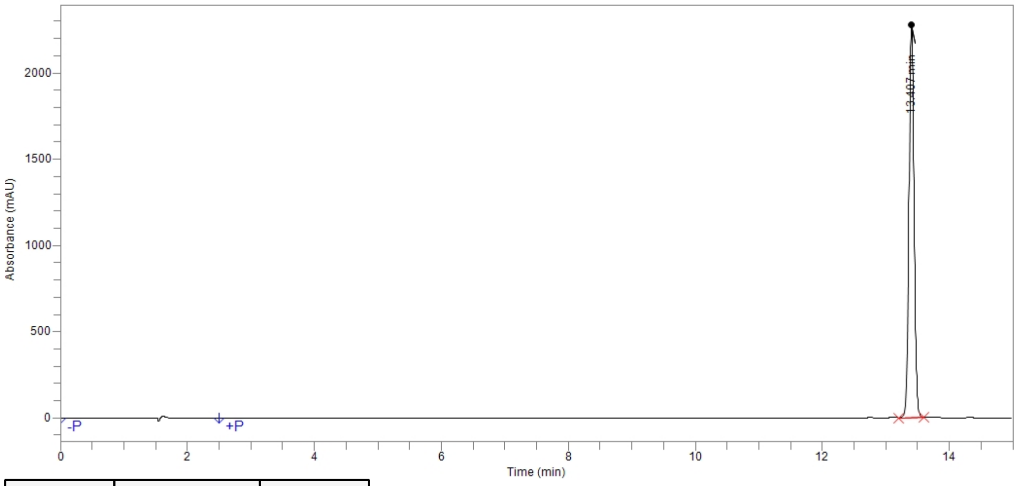

Time	Area	Area %
13.407	13,108,258.3	100.00
**Total**	13,108,258.3	100.00

(*E*)-*N*-(4-(3-((4,5-Dimethoxy-2-nitrobenzyl)oxy)-3-oxoprop-1-en-1-yl)benzyl)-2-(2-methyl-1*H*-indol-3-yl)ethan-1-aminium trifluoroacetate (**1**, Zap-Pano·TFA)

EC5-16 **2/4/2019**

Acquisition Method	Purity short run @254 nm
Acquisition Date/Time	2/4/2019 4:27 pm
Injection Volume	10
Sample Name	EC5-16
Sample Description	
Batch Description	For start up

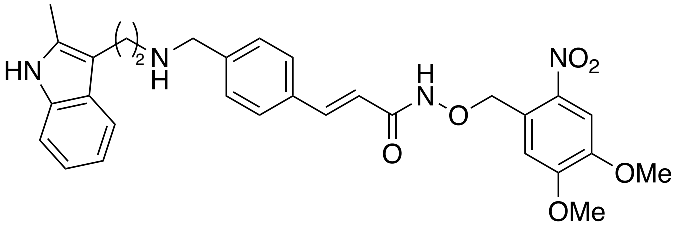

EC5-16 : Injection 1

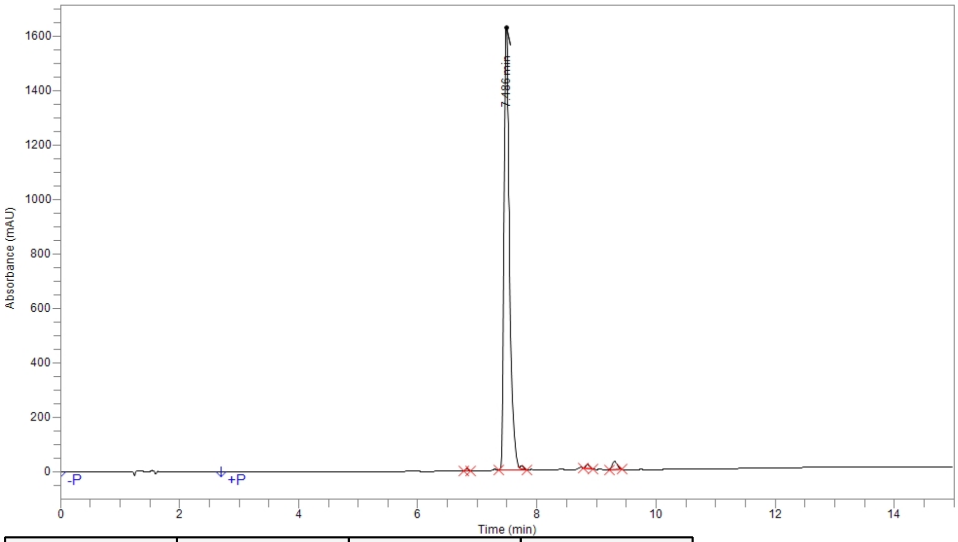

Time	Height	Area	Area %
6.826	6,818.0	25,051.6	0.23
7.486	1,624,206.1	10,592,235.0	97.20
7.746	12,212.0	51,254.2	0.47
8.849	15,436.2	67,951.5	0.62
9.303	29,285.4	160,642.4	1.47
**Total**		10,897,134.7	100.00

(*E*)-3-(4-(((2-(2-Methyl-1*H*-indol-3-yl)ethyl)amino)methyl)phenyl)prop-2-enamide **(15)**

EC2-49 **Purity short run @254 nm** **3/7/2017 10:13 am**

Sample Name EC2-49
Vial Number 1
Injection Volume 10
Acquisition Date/Time 3/7/2017 9:56 am
Acquisition Method Purity short run @254 nm
Processing Method Purity short run @254 nm

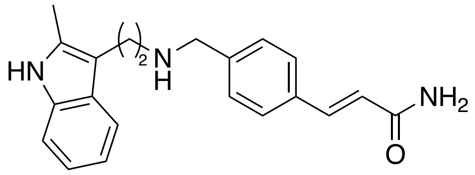

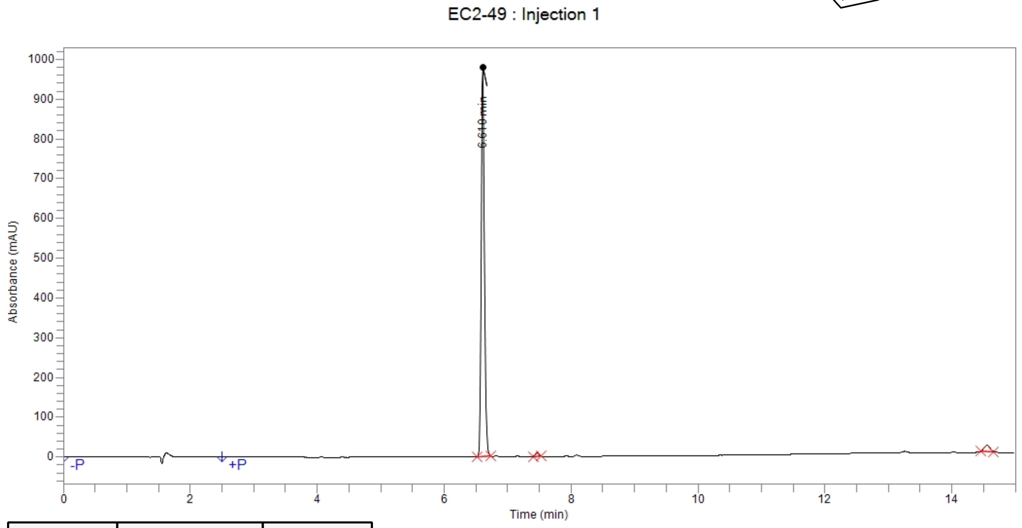

Time	Area	Area %
6.610	3,324,959.2	96.21
7.462	33,716.5	0.98
14.555	97,152.7	2.81
**Total**	3,455,828.4	100.00

3/7/2017 10:13 am **Flexar HPLC 2**

LCMS comparison of Pano-NH_2 (**15**) and Pano acid

Preparative LC - With MS Spectra
PANO acid_PANO amide SPIKE

Agilent

**Data file:**	C:\Users\Public\Documents\ChemStation\1\Data\PANO acid_DSCH05051 2021-07-02 06-47-46\003-P1-E11-PANO acid_PANO amide SPIKE.D		
**Sample name:**	PANO acid_PANO amide SPIKE		
**Description:**			
**Injection:**	1 of 1	**Location:**	P1-E11
**Injection volume:**	10.000	**Sample amount:**	0.000
**Instrument:**	LCMS	**Injection date:**	2021-07-02 07:20:50+00:00
**Acq. operator:**	SYSTEM	**Acq. method:**	ZAP-PANO_MECN_20_80_H2O_FA_100_1000_POS_NEG.FULL.M
**Analysis method:**	ZAP-PANO_MECN_20_80_H2O_FA_100_1000_POS_NEG.FULL.M	**Last changed:**	2021-07-02 07:11:00+00:00

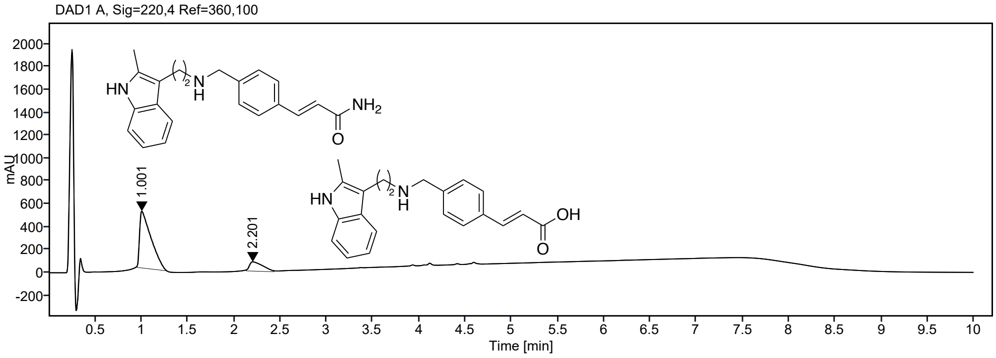

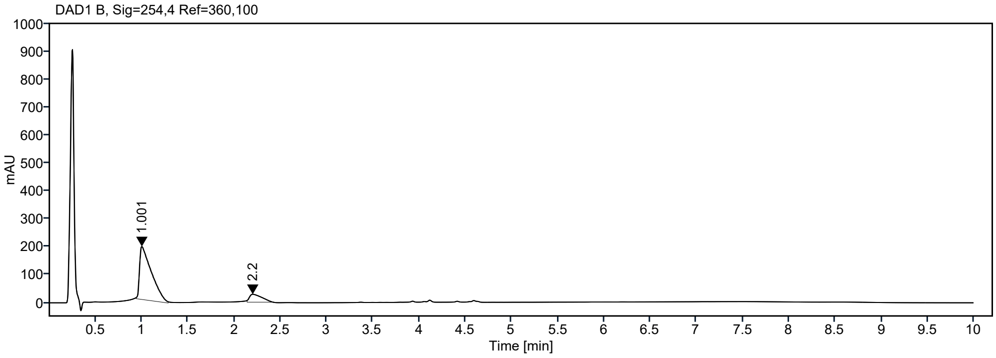

DAD1 C, Sig=280,4 Ref=360,100

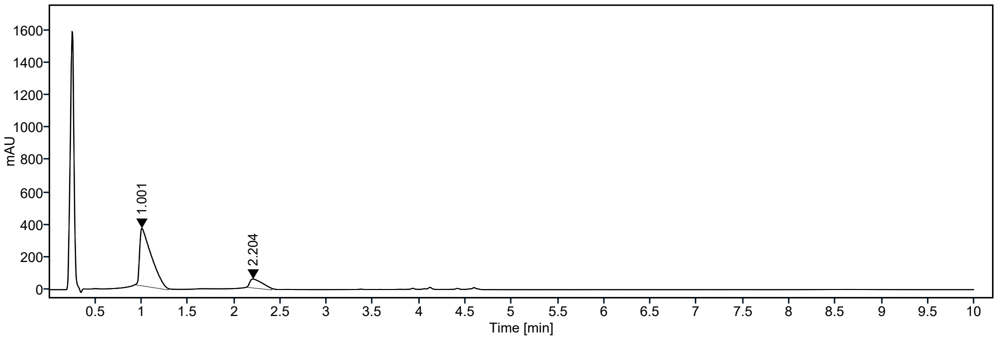

MSD1 TIC, MS File, ES-API, Pos, Scan, Frag: 70, "Positive"

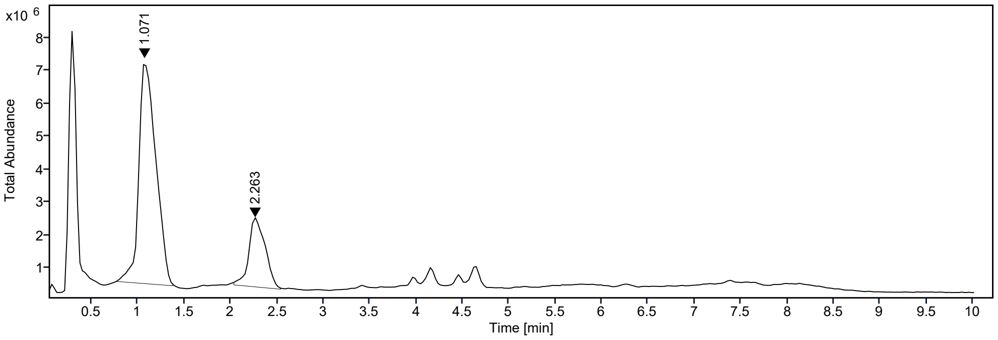

MSD2 TIC, MS File, ES-API, Neg, Scan, Frag: 70, "Negative"

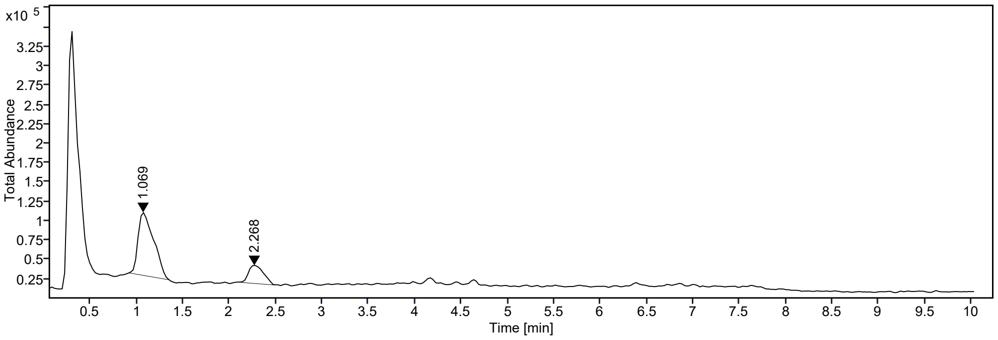

Signal details: MSD1 TIC, MS File, ES-API, Pos, Scan, Frag: 70, "Positive"

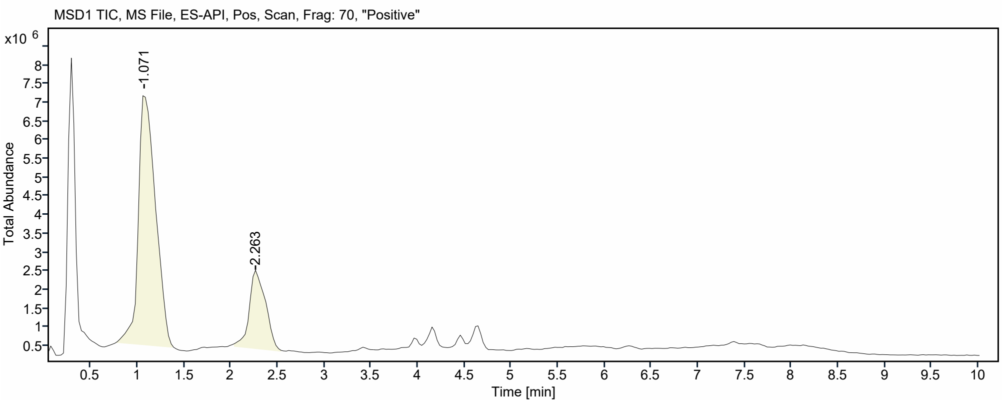

RT [min]	Signal Description	Symmetry	Resolution	Height [count]	Area [count*min]	Rel. Area [%]
1.071	MSD1 TIC, MS File, ES-API, Pos, Scan, Frag: 70, "Positive"	0.51739		6858366	86063536	77.0
2.263	MSD1 TIC, MS File, ES-API, Pos, Scan, Frag: 70, "Positive"	0.62214	3.72645	2113574	25744076	23.0

Filtered on peak height > 100000 counts

Retention time: 1.071 min **Area Percent:** 77%

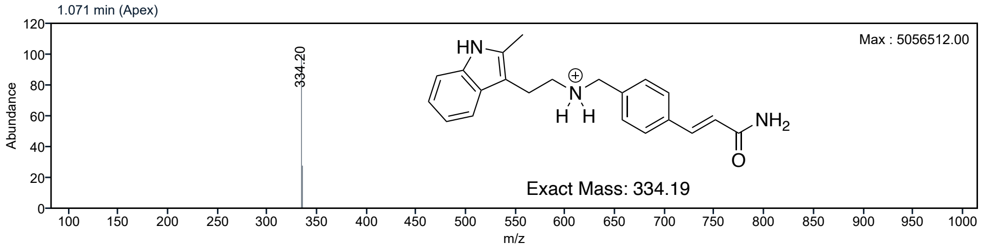

Retention time: 2.263 min **Area Percent:** 23%

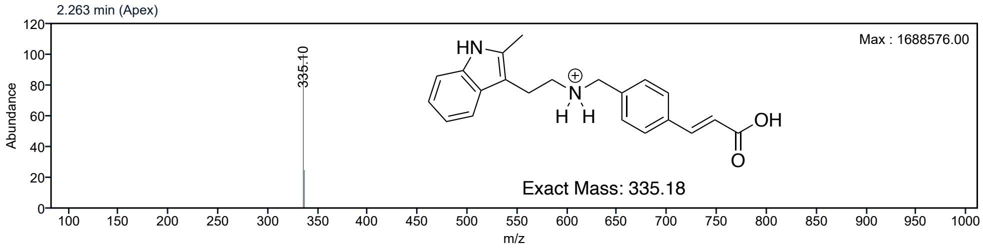

**Acquisition Method:**	**ZAP-PANO_MECN_20_80_H2O_FA_100_1000_POS_NEG.FULL.M**
**Path:**	**C:\Users\Public\Documents\ChemStation\1\Data\PANO acid_DSCH05051 2021-07-02 06-47-46**
**Acquisition Method:**	**ZAP-PANO_MECN_20_80_H2O_FA_100_1000_POS_NEG.FULL.M**
**Path:**	**C:\Users\Public\Documents\ChemStation\1\Data\PANO acid_DSCH05051 2021-07-02 06-47-46**
**Data Analysis Method:**	**ZAP-PANO_MECN_20_80_H2O_FA_100_1000_POS_NEG.FULL.M**
**Path:**	**C:\Users\Public\Documents\ChemStation\1\Data\PANO acid_DSCH05051 2021-07-02 06-47-46**

Method Information

Method:	C:\Users\Public\Documents\ChemStation\1\Data\PANO acid_DSCH05051 2021-07-02 06-47-46\ZAP-PANO_MECN_20_80_H2O_FA_100_1000_POS_NEG.FULL.M
Modified:	1/1/1970 at 12:00:00 AM
Description:	80%H2O/20%MeCN +0.1% FA to 30%H2O/70%MeCN + 0.1% FA over 6 minutes, 40 °C
